# Neural transcriptomic signature of chronic wasting disease in white-tailed deer

**DOI:** 10.1186/s12864-022-08306-0

**Published:** 2022-01-21

**Authors:** Eóin O’Hara, Allen Herbst, Arun Kommadath, Judd M. Aiken, Debbie McKenzie, Negin Goodarzi, Pamela Skinner, Paul Stothard

**Affiliations:** 1 Department of Agricultural, Food and Nutritional Science, University of Alberta, 2-31 General Services Bldg, Edmonton, AB T6G 2H1 Canada; 2 Lethbridge Research and Development Centre, Agriculture and Agri-Food Canada, Lethbridge, Alberta Canada; 3 Lacombe Research and Development Centre, Agriculture and Agri-Food Canada, Lacombe, Alberta Canada; 4 Department of Biological Sciences, University of Alberta, Edmonton, Alberta Canada; 5 Department of Veterinary and Biomedical Sciences, University of Minnesota, St. Paul, MN USA

## Abstract

**Background:**

The increasing prevalence and expanding geographical range of the chronic wasting disease (CWD) panzootic in cervids is threatening human, animal, environmental and economic health. The pathogenesis of CWD in cervids is, however, not well understood. We used RNA sequencing (RNA-seq) to compare the brain transcriptome from white-tailed deer (WTD; *Odocoileus virginianus*) clinically affected with CWD (*n* = 3) to WTD that tested negative (*n* = 8) for CWD. In addition, one preclinical CWD+ brain sample was analyzed by RNA-seq.

**Results:**

We found 255 genes that were significantly deregulated by CWD, 197 of which were upregulated. There was a high degree of overlap in differentially expressed genes (DEGs) identified when using either/both the reference genome assembly of WTD for mapping sequenced reads to or the better characterized genome assembly of a closely related model species, *Bos taurus*. Quantitative PCR of a subset of the DEGs confirmed the RNA-seq data. Gene ontology term enrichment analysis found a majority of genes involved in immune activation, consistent with the neuroinflammatory pathogenesis of prion diseases. A metagenomic analysis of the RNA-seq data was conducted to look for the presence of spiroplasma and other bacteria in CWD infected deer brain tissue.

**Conclusions:**

The gene expression changes identified highlight the role of innate immunity in prion infection, potential disease associated biomarkers and potential targets for therapeutic agents. An association between CWD and spiroplasma infection was not found.

**Supplementary Information:**

The online version contains supplementary material available at 10.1186/s12864-022-08306-0.

## Introduction

Chronic wasting disease (CWD) is a naturally occurring, universally fatal prion disease or transmissible spongiform encephalopathy (TSE) of cervids (e.g. deer, moose, elk) [1]. First described in captive Colorado mule deer in 1967, the disease is now found in farmed and free-ranging cervid populations across North America, with additional cases documented in Scandinavia and South Korea [2]. The prevalence of CWD can be very high, above 50% in some free-ranging populations and 90% in some captive herds. Surveillance and management of captive cervids is effective in reducing disease prevalence [2]. Continued expansion of endemic areas may have significant implications for the long-term sustainability of cervid populations in North America [3].

TSEs are progressive neurodegenerative disorders, characterised by the aggregation of misfolded prion proteins in the neural tissues of infected hosts [4]. The infectious prion, PrP^CWD^, converts the cellular prion protein, PrP^C^, into PrP^CWD^. Migration of the infectious prions to the nervous system results in full brain involvement and clinical disease and involves widespread deposition of PrP^CWD^ in numerous brain areas, but especially the obex, superior colliculus, hypothalamus, septal nucleus of the basal ganglia and cerebellum [5]. The accumulation of PrP^CWD^ leads to gliosis and astrogliosis, hallmark neuronal vacuolation, progressive degeneration, and ultimately, death of the host [6].

Increasing our understanding of the key pathways affected by the accumulation of PrP^CWD^ may provide targets for early disease diagnosis or transmission and enable approaches to mitigate the disease. A previous study of neural transcript expression profiles in BSE-infected macaques indicated the dysregulation of genes involved in oxygen and lipid transport, and in innate immune processes [7]. Basu and colleagues similarly reported that CWD-infected elk had altered expression of immune-regulatory gene transcripts in the brain [8]. A recent study in deer utilized high-throughput sequencing to examine the impact of CWD on transcription profiles in liver and lymph node tissues [9], but to the best of our knowledge, no study has used RNA sequencing (RNA-seq) to examine neural gene expression profiles in CWD-infected cervids. In the present study, we use RNA-seq of brain tissue collected from CWD-positive and CWD-negative white-tailed deer (WTD; *Odocoileus virginianus*) to assess the transcriptome-level response to the disease. Compared to the previous bovine microarray-based study of brain gene expression in CWD-infected elk, our study has the opportunity to assess a broader range of transcripts while avoiding the detection issues associated with probes that reduce performance for identifying differentially expressed genes [10, 11]. The findings detailed here contribute to increasing our understanding of the molecular mechanisms underlying CWD-induced neurotoxicity in cervids.

## Materials and methods

### Deer brain samples

Deer brain tissue was obtained at necropsy from one uninfected and three clinically-affected WTD that had been orally infected with Wisc-1 prions [12]. Additional deer brain samples were obtained from hunter-harvested WTD (Wisconsin, CWD test negative, *n* = 3) and from a Saskatchewan white-tail deer herd depopulated for CWD control (*n* = 4). One deer from Saskatchewan was test positive for CWD (NBSK23); the other three were test-negative. All CWD+ deer were positive in both the obex and the retropharyngeal lymph nodes. Brain tissue from all deer were removed within 2 h of death, bisected along the sagittal plane and frozen on dry ice. Brain tissue did not include the obex region of the medulla oblongata which was used for CWD testing. Brain hemispheres were homogenized to a powder under liquid nitrogen using a mortar and pestle, and the total brain powder was sampled for subsequent RNAseq analysis. All the samples were from adult deer. The sex of some of those deer were unknown, so those were determined based on mapping of RNA-seq reads from those samples to the sequence of the bovine Y chromosome. The CWD status of deer was determined by the Wisconsin Department of Natural Resources or the Canadian Food Inspection Agency using immunochemical detection of CWD prions in the retropharyngeal lymph nodes andr immunohistochemical staining of the obex region of the medulla oblongata.

### Western blot analysis

Powdered brain tissue was homogenized in an Omni Bead ruptor (Omni International, Kennesaw Georgia) with cold sterile water (10% w/v). A subset of brain homogenates were digested with proteinase K (50 mg/ml) for 30 min at 37 °C, boiled in Laemmli buffer for 10 min, fractionated on 12% NuPAGE Bis-tris gels (Invitrogen) and transferred to PVDF membrane (Millipore). Membranes were blocked in 0.1% TBS-T / 5% milk for 1 h at room temperature. Incubation with the primary antibody, Bar224 1:10,000 (Cayman Chemical), was performed overnight at 4 °C. Anti-mouse IgG alkaline phosphatase conjugated secondary antibody (Promega) was used at 1:10,000 with a fluorescent substrate (Attophos Promega).

### RNA purification, library preparation and sequencing

Total RNA was isolated from powdered brain tissue using a commercial kit, following the manufacturer’s instructions (RNeasy Mini, Qiagen). RNA quality was assessed via spectrophotometry (NanoDrop Technologies, Inc.) and gel electrophoresis (Bioanalyzer 2100, Agilent Technologies). Library construction and sequencing were performed by the Centre d’Expertise et de Services of Génome Québec. Libraries were generated from 250 ng of total RNA. Messenger RNA enrichment was performed using the NEBNext Poly(A) Magnetic Isolation Module (New England BioLabs). cDNA synthesis was achieved with the NEBNext RNA First Strand Synthesis and NEBNext Ultra Directional RNA Second Strand Synthesis Modules (New England BioLabs). The remaining steps of library preparation were completed using the NEBNext Ultra II DNA Library Prep Kit for Illumina (New England BioLabs). Libraries then underwent 2x100bp sequencing using an Illumina HiSeq2500 platform. Sequencing data have been deposited into NCBI, accession number PRJNA756812.

### Sequence read alignment to reference genomes

Following sequencing, raw reads were inspected for quality using FastQC v0.11.9 [13] and MultiQC [14]. Trimmomatic v0.39 [15] was used to remove poor quality (q < 33) and short (< 36 nt) reads. The latest versions of the WTD reference genome assembly (Ovir.te.1.0) and its associated gene annotation file were downloaded from NCBI (GenBank assembly accession: GCF_002102435.1). Prior to analysis, 25 rows in the GTF annotation file with an empty ‘gene_id’ attribute field and with gbkey “tRNA” were removed. The STAR short read aligner v2.7.3a [16] was used to index the reference genome and for subsequent alignment of the filtered read set. Mapping results were obtained in BAM format, sorted using samtools v1.7.0 [17] and used as input for featureCounts v2.0.0 [18] to count the number of alignments that overlapped exonic regions on both the sense and antisense strands, separately. Gene count matrices were exported for downstream analyses in R v3.6.1 [19].

Sequence reads were also mapped to the most recent bovine reference genome assembly (ARS-UCD1.2; Ensembl Release 100, April 2020) with the expectation that the more complete genome sequence and annotations could provide additional insights into gene expression changes, using the read mapping and counting strategy as described above. To determine/verify the sex of the deer, the reads that remained unmapped after the initial alignment to the bovine reference genome (lacking the Y chromosome) were mapped to a separate bovine Y chromosome sequence (NCBI GenBank ID: CM011803.1) using STAR aligner and the counts of reads assigned to gene features on the Y chromosome were determined using featureCounts. Similar approaches have been used previously [20]. The ratio of the total reads that remained unmapped to any gene features on the bovine Y chromosome over the total reads (assigned and unassigned) based on the featureCounts summary report was then calculated. The distribution of those ratios were bimodal with known female deer having a ratio below 0.04 and known male deer having a ratio above 0.08. A Welch two-sample t-test to test for difference between means of the 2 groups indicated a highly significant difference (*p*-value was 1.922e-15), with the mean of the female group at 0.0293 (SD 0.0022) and the male at 0.0857 (SD 0.0024)). Sex determined by sequencing was 100% accurate with those deer of known sex.

### Data exploration and differential gene expression

The steps followed for downstream analysis were identical for read counts generated from read mapping to WTD as well as to bovine genome reference sequences. Prior to downstream analysis, genes with low or inconsistent read depth (less than 15 reads in total across all samples, and/or less than 10 reads mapped per sample) were filtered out. A power analysis conducted using RNASeqPower version 1.26.0 [21] showed that with our relatively modest sample sizes, gene expression fold changes of 1.75 could be reliably detected at a power of 0.8 and a fixed false discovery rate (FDR) of 0.05. Normalized gene counts were fitted to a generalized linear model to identify differentially expressed genes (DEGs) according to CWD status. The model also accounted for variability arising between sequencing batches. Data was normalized using the trimmed mean of M-values procedure implemented in edgeR to account for technical variation between samples [22], and DEGs were identified using the thresholds outlined above. The preclinical sample (NBSK23) was not included in the differential expression analysis, maximizing the contrast between infected and healthy samples.

### Enrichment analysis of differentially expressed genes

Functional enrichment analysis was performed using DAVID v6.8 to identify Gene Ontology (GO) terms (Biological Processes, Molecular Functions, Cellular Components) over-represented in the DEGs against a population set consisting of all genes identified as expressed [23, 24]. Only terms containing at least 5 DEGs were deemed enriched in our dataset at a threshold of FDR-adjusted *P* < 0.05. DEGs from the bovine reference genome mapping study were used as DAVID knowledgebase does not have GO mappings to WTD gene IDs, and the bovine genome is better annotated.

### Quantitative real-time PCR validation

Total RNA from WTD brain tissues was used for quantitative real-time PCR (qRT-PCR). RNA from the three CWD positive deer, four CWD negative deer, and one preclinical CWD positive deer was reverse transcribed to cDNA using TaqMan Reverse Transcription Reagents kit (Applied Biosystems). A CFX96 Real-Time instrument (Bio-Rad) and iTaq Universal SYBR Green Supermix (Bio-Rad) were used following the manufacturer’s instructions. PCR primers were designed using Primer Blast [25]. PCR Primers (Supplementary Table 1, Additional File 1) either spanned introns or included an exon junction. The annealing temperature was 58 °C for all reactions. Pooled cDNA prepared from white-tailed deer lymph nodes and brain was used to optimize primers and to produce standard curves. A reaction without template was used as a negative control for each target. The relative cDNA levels were calculated using the 2^-ΔΔCt^ method, with β-actin used as the reference gene for normalization as previously described [26, 27]. A two-sample t-test with unequal variance was performed on the data for each target. The back-transformed ratios (2^-ΔΔCt^), along with 95% CI and *p*-values, are reported. To assess evidence for any overall difference between the positive and negative groups across all target genes, a linear mixed model was fitted with fixed effects for target and the group/target interaction and a random effect for sample, where the group/target interaction is the term that assesses differences between the groups across the targets.

### Investigation of the neural bacteriome using Kraken2

Reads that failed alignment to the Ovir.te.1.0 genome were mapped to a database comprising the entire collection of complete bacterial genomes deposited in NCBI using Kraken2 (v.2.1.1) [28] (database created in Dec. 2020), to assign taxonomic tags to all recovered bacterial sequences. Following read mapping, genomes represented by < 2 reads across all samples were removed. DeSeq2 was used to identify differentially detected bacteria (FDR < 0.05) according to disease status. Sequencing batch effects were included in the generalized linear model. All plots depicting microbial data were prepared in R using base R packages and *ggplot2*.

## Results

### PrP^CWD^ abundance in the brains of white-tailed deer

**Fig. 1:**
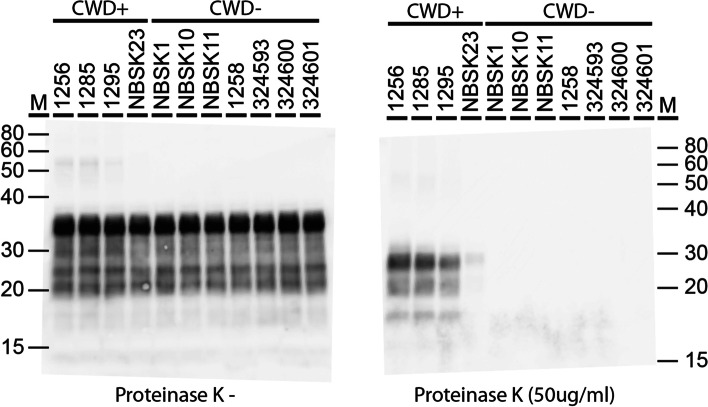
Western blot analysis of prion protein from deer brain samples used for transcriptional profiling. Powdered brain tissue was homogenized to 10% w/v in water and analyzed by Western blotting using the monoclonal antibody Bar224 at a dilution of 1:10,000. Prion proteins were examined with and without proteinase K treatment. The NBSK23 sample is from a preclinical infection. Standardized amounts of brain homogenate based on volume were loaded in each lane

Differential gene expression induced by CWD will be affected by the stage of prion infection as determined by prion neuroinvasion and accumulation in the central nervous system. The three clinically affected deer had high levels of proteinase-K resistant PrP isoforms (PrP^CWD^) in their brains as determined by western blot (Fig. 1). One deer (NBSK23) was culled at a preclinical time point and possessed a lower abundance of PrP^CWD^. The time post-infection is unknown.

### Characteristics of RNA-Seq data

**Fig. 2:**
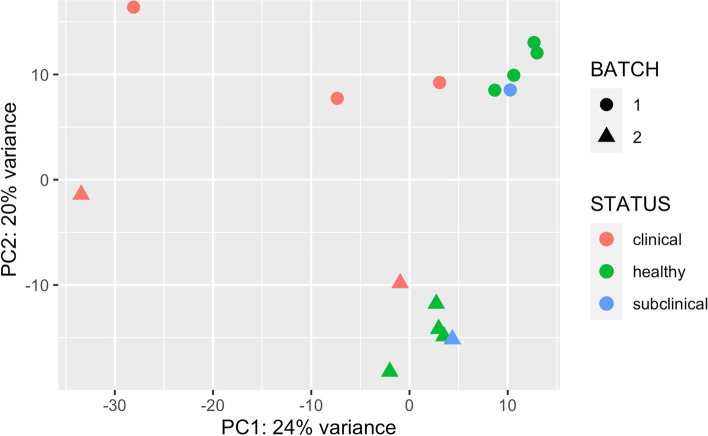
Principal component analysis (PCA). Gene expression data from each sample was transformed by projecting each measure of expression onto two principal components. Count data was subjected to variance stabilizing transformation prior to PCA plot construction using the DeSeq2 and ggplot2 R Bioconductor packages

RNA used for sequencing library prep was of high quality (RIN > 7), even in the hunter harvest samples. RNA sequencing generated an average of 46.5 ± 7.05 million reads per sample. Descriptive details of samples, sequencing data and mapping characteristics are provided in Table 1. Of interest, the preclinical animal (NBSK23) did not cluster closely with the clinically infected deer in either the PCA plot (Fig. 2) or the hierarchical clustering (Supplementary Fig. 1, Additional File 1). Greater inter-animal variation in gene expression profiles was evident among CWD affected deer than the healthy cohort. The sequencing data, in general, differed according to batch in terms of alignment rate. On average, the total number of sequencing reads in batch one was 22% higher than from batch two.

**Table 1:** Details of sample metadata and RNA-seq analysis

Sample Metadata				Sequencing		Post Quality Control		White-Tailed Deer Reference Mapping				Bovine Reference Mapping			
Sample ID	CWD Status	Technical replicate	RIN	Batch	reads (M)	reads (M)	reads (%)	Unique alignments		Assigned to genes		Unique alignments		Assigned to genes	
								(M)	(%)	(M)	(%)	(M)	(%)	(M)	(%)
1256-1c	+	No	8.9	1	56.2	51.8	92.1	48.6	93.9	38.8	73.4	38.2	73.8	30.4	73.0
1258-1 h	–	No	8	1	55.9	51.6	92.4	48.4	93.7	38.3	72.6	37.7	73.0	30.0	72.7
1285-1c	+	No	7.8	1	53.4	49.4	92.6	46.2	93.5	36.0	71.3	35.4	71.6	28.1	72.3
1295-1c	+	No	7.7	1	50.3	46.6	92.5	43.6	93.7	34.1	72.1	33.6	72.3	26.8	72.7
324,593-1 h	–	No	7.8	1	40.6	37.5	92.4	35.2	93.8	27.6	72.1	27.2	72.5	21.8	73.3
324,600-1 h	–	No	7.9	1	57.8	53.4	92.4	50.0	93.7	39.0	71.7	38.4	71.9	30.9	73.5
NBSK10-1 h	–	No	7.2	1	49.4	45.7	92.5	42.7	93.5	33.0	70.9	32.6	71.3	25.7	72.0
NBSK23–1 s	preclinical	No	7.4	1	43.1	39.7	92.1	37.1	93.5	28.4	69.9	28.1	70.6	22.0	71.6
1285-2c	+	Yes	7.8	2	33.0	22.2	67.3	20.5	92.6	15.6	68.3	15.0	67.7	11.7	69.9
1295-2c	+	Yes	7.7	2	47.3	33.2	70.2	30.8	92.9	24.2	71.2	23.3	70.2	18.2	70.7
324,601-2 h	–	No	7.3	2	44.0	31.6	71.8	29.1	92.3	22.0	67.7	21.4	67.6	16.9	71.3
NBSK1-2 h	–	No	7.4	2	46.9	34.7	74.0	32.2	92.9	25.1	70.7	24.3	70.2	19.2	71.4
NBSK11-2 h	–	No	8.2	2	41.7	29.8	71.5	27.8	93.0	21.9	71.5	21.2	70.9	16.7	71.6
NBSK23–2 s	preclinical	Yes	7.4	2	38.3	26.9	70.3	24.9	92.7	19.0	68.9	18.5	68.8	14.4	70.4
NBSK27-2 h	–	No	NA	2	40.3	29.5	73.2	27.3	92.6	21.3	70.7	20.7	70.3	16.3	70.9

### Identification of differentially expressed genes

**Fig. 3:**
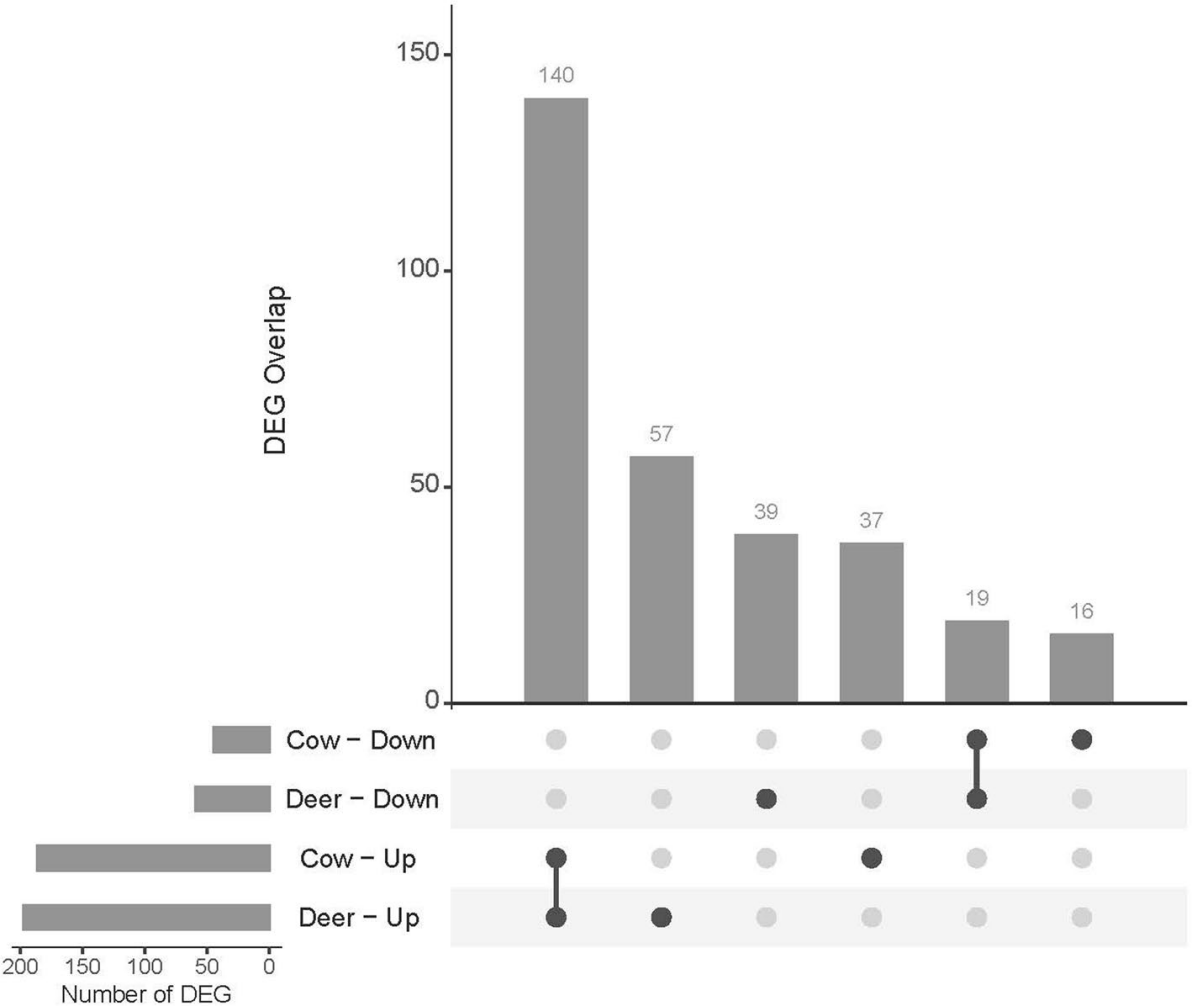
Upset plot showing the numbers and interactions of differentially expressed genes (DEG) detected using edgeR on count data generated from alignment against both the cow and deer reference assemblies. The horizontal bars show the number of DEG in each set (up = up-regulated, down = down-regulated), while the vertical bars represent the intersection size. Dark spheres indicate which sets are represented in the vertical bar

Differential gene expression analysis using edgeR identified 255 genes that were significantly affected by CWD infection when mapped to the WTD genome assembly. 197 genes were upregulated in clinically affected CWD+ deer. A subsequent analysis using mapping to the bovine reference assembly identified 229 DEGs, with 186 of these upregulated in the clinically infected animals (Additional File 2). There was significant overlap between the sets of DEGs identified by mapping to either the WTD or bovine genome (Fig. 3). 140 upregulated genes and 19 downregulated genes were common to both datasets. Unannotated genes (denoted with “LOC” symbol preceding NCBI’s Entrez GeneID) comprised 27.05% of the DEGs identified using the WTD reference assembly. In contrast, just 6.68% of the bovine DEGs lacked an Ensembl annotation (denoted “NA”).

### Functional enrichment of differentially expressed genes

**Fig. 4:**
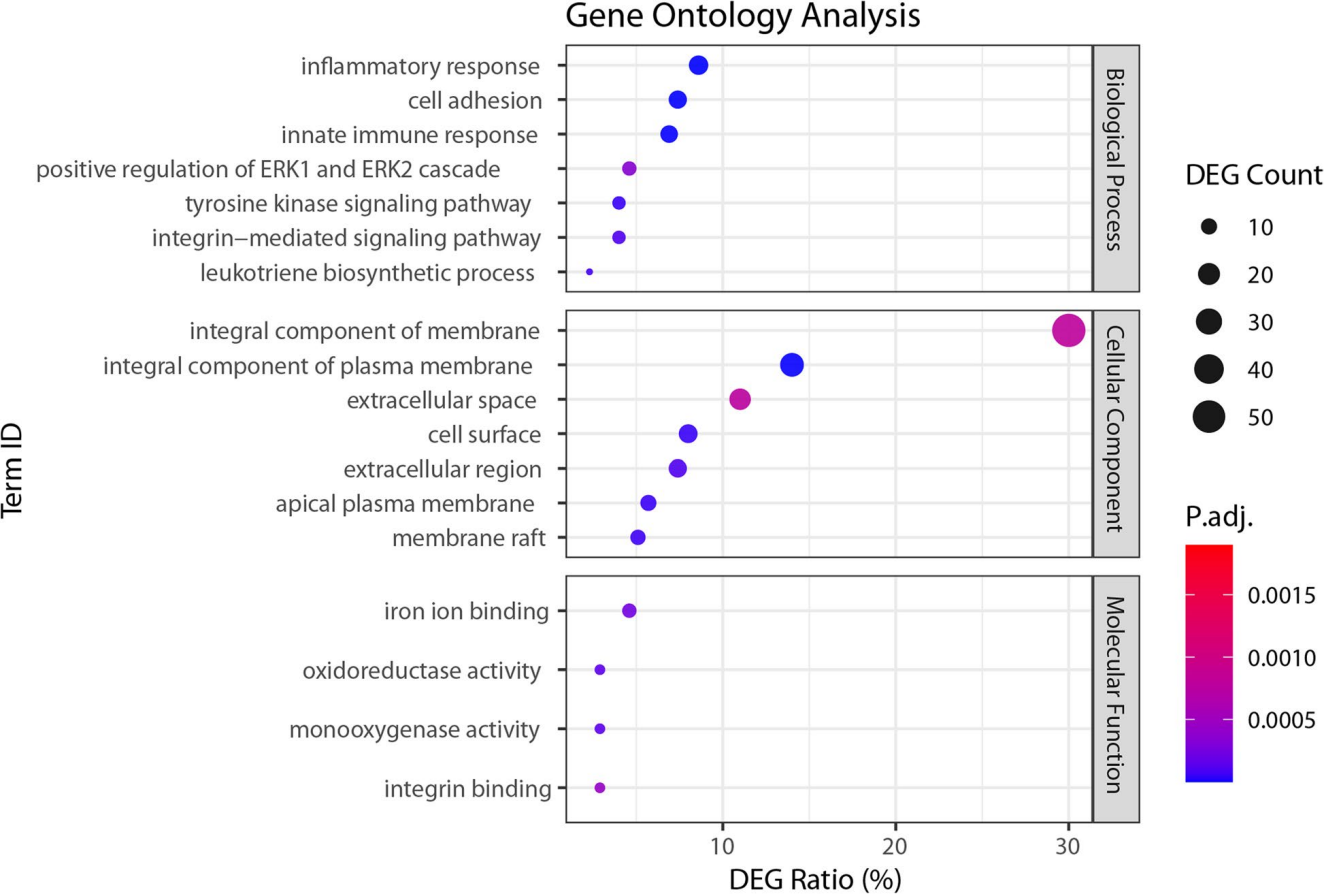
Bubble plots indicating the functional enrichment profiles among the DEG upregulated in CWD+ animals. Data are stratified by term group and ranked according to Benjamini Hochberg-adjusted *P*-value. The size of the bubble indicates the number of DEG contained in the enriched term. Analysis was performed using the set up upregulated genes obtained from mapping to the bovine genome due to the relatively large number of unannotated DEG detected using the deer genome

Enrichment analysis was performed for the DEGs identified using the bovine reference genome, as the bovine gene annotations (Ensembl gene IDs used) allowed for more complete GO term assignment (terms were assigned for 186 upregulated and 34 downregulated DEGs). Eighteen GO terms (7 Biological Processes, 7 Cellular Component, 4 Molecular Function) were over-represented among the upregulated genes (adj. *P* < 0.05, Fig. 4, Table 2). The Biological Processes enriched in CWD+ brain tissue included GO:0006954 – inflammatory response, GO:0045087 – innate immune response, GO:0007229 – integrin-mediated signaling pathway and GO:0045087 – Phagosome. Molecular Functions enriched among the DEGs detected in CWD+ brain tissue included GO:00004497 – monooxygenase activity, GO:0005506 iron ion binding, and GO:0005178 – integrin binding. No enriched terms were identified among the downregulated DEGs.

**Table 2:** Gene ontology pathway terms enriched among the DEG

Term		DEG (%)^1^	Fold Enrichment	*P*-value^2^
**Biological Process**				
GO:0006954	inflammatory response	8.6	8.2	< 0.001
GO:0007155	cell adhesion	7.4	6.5	< 0.001
GO:0045087	innate immune response	6.9	6.2	0.001
GO:0007169	transmembrane receptor protein tyrosine kinase signaling pathway	4.0	10.0	0.011
GO:0019370	leukotriene biosynthetic process	2.3	41.0	0.015
GO:0007229	integrin-mediated signaling pathway	4.0	9.0	0.016
GO:0070374	positive regulation of ERK1 and ERK2 cascade	4.6	6.0	0.042
**Cellular Component**				
GO:0005887	integral component of plasma membrane	14.0	3.1	< 0.001
GO:0009986	cell surface	8.0	3.9	0.004
GO:0016324	apical plasma membrane	5.7	5.7	0.003
GO:0045121	membrane raft	5.1	6.5	0.003
GO:0005576	extracellular region	7.4	3.9	0.003
GO:0005615	extracellular space	11.0	2.4	0.019
GO:0016021	integral component of membrane	30.0	1.5	0.017
**Molecular Function**				
GO:0004497	monooxygenase activity	2.9	18.0	0.034
GO:0016705	oxidoreductase activity, acting on paired donors, with incorporation or reduction of molecular oxygen	2.9	18.0	0.034
GO:0005506	iron ion binding	4.6	6.4	0.027
GO:0005178	integrin binding	2.9	13.0	0.035

^1^The number of DEG present in the list of upregulated genes as a percentage of the total number of genes involved in each respective term or pathway. ^**2**^P-values are Holm-Bonferroni corrected

### Validation of gene expression profiles

**Fig. 5:**
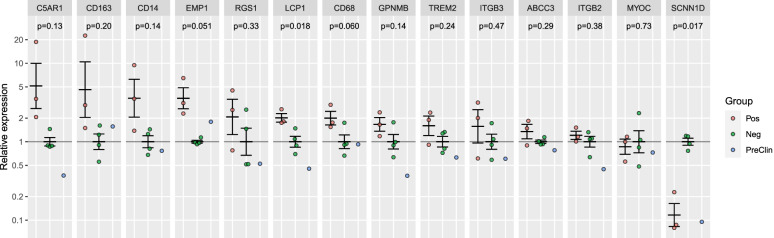
Fourteen genes de-regulated by CWD were validated by RT-qPCR. Relative gene expression levels for each gene are presented for the CWD positive (red), CWD negative(green) and preclinical(blue) groups. A relative gene expression method (2^-ΔΔCt^) was used to normalize and compare the data from each group. The error bars show the mean +/− SE

Quantitative real-time PCR was used to further evaluate fourteen DEGs identified by the RNA-seq analysis. Consistent with the RNA-seq results, we found that *MYOC* and *SCNN1D* were downregulated and that *ABCC3*, *C5AR1*, *CD14*, *CD68*, *CD163*, *EMP1*, *GPNMB*, *ITGB2*, *ITGB3*, *LCP1*, *RGS1*, *TREM2* were upregulated. The relative expression levels of each gene are presented in Fig. 5. A mixed model analysis showed statistically significant evidence for overall differences between the infected and uninfected deer across all target genes; the group/target interaction was statistically significant, with *p* < 0.0001. At the level of individual genes, statistical power was limited by the sample number and the variation, especially in the CWD+ samples. Unadjusted *p*-values calculated using student’s t-test are reported for each gene.

### Bacterial sequence provenance in deer brain tissue

**Fig. 6:**
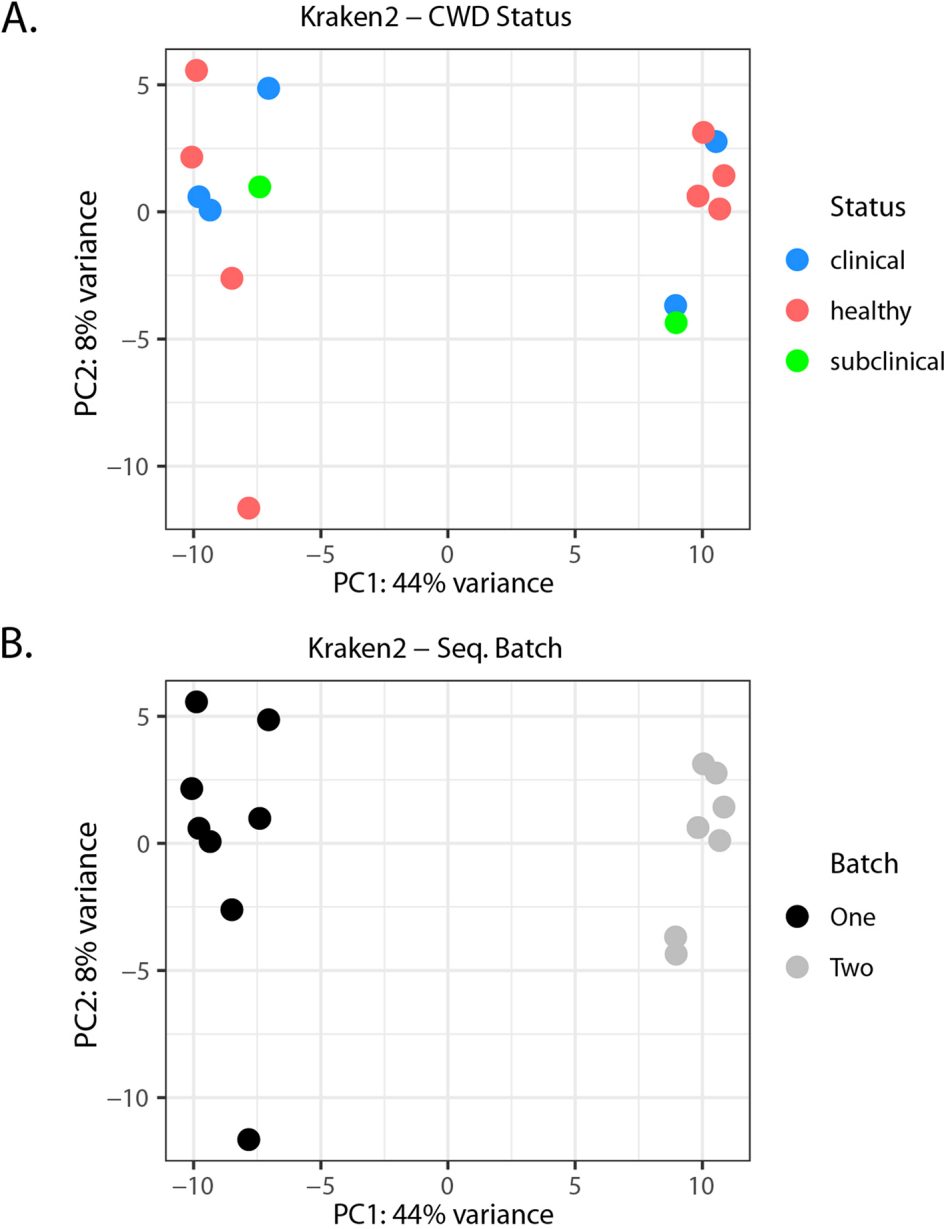
Lack of bacterial signature in CWD affected deer. Principal component analysis plots showing the samples of neural tissue according to (**A**.) disease status and (**B**.) sequencing batch generated using Kraken2 following alignment of all non-deer reads to a reference database comprising all completed NCBI bacterial genomes

An alternative hypothesis to the prion hypothesis suggests that CWD is the result of a Spiroplasma infection [29]. We tested this hypothesis by analyzing our sequencing data to assess the taxonomic provenance of bacterial sequences recovered, and investigating whether this microbial community had a signature related to CWD status. We reasoned that if spiroplasma bacteria contributed to CWD pathogenesis, then we should be able to detect these transcripts in a CWD specific manner. We mined our data, by aligning the non-deer reads to a database of complete bacterial genomes, to determine the nature, if any, of bacterial expression in the neural tissues of deer. Approximately 17,000 non-deer reads per sample were aligned to a database of complete bacterial genomes using Kraken2, which mapped to 463 non-singleton bacterial species. Principal component analysis did not separate the data according to disease status (Fig. 6). These data do not support a hypothesized bacterial etiology for CWD. Rather, the sequencing batch was the major determinant of differences in the reads mapped to microbial communities (Fig. 6, Supplementary Fig. 2, Additional File 1). The most abundant reads that mapped to the bacterial database were to transcripts from *Staphylococcus haemolyticus*, which contributed, on average, 10.8% of all bacterial-mapped reads per sample. Other bacterial-mapped reads aligned with transcripts from *Oceanobacillus iheyensis, Streptomyces lavendulae,* and *Escherichia coli*. Less than 0.00008% of the reads mapped to genes from *Spiroplasma* species, about 38 reads. Differential abundance analysis of bacterial-mapped reads detected using DeSeq2 did not reveal any significantly different abundance between clinical and healthy animals (FDR > 0.05), in a model that included sequencing batch, and excluded the subclinical animal.

## Discussion

In the face of ecological, economical and, potentially, public health risks of CWD, it is important to gain further understanding of disease pathogenesis on a molecular scale. Transcriptional regulation is a primary mechanism underpinning disease progression [30], but the limited data published on CWD-infected cervids has employed microarray analysis of lymphatic and neural elk tissues [8] and RNA-seq of lymphatic and hepatic samples collected from infected deer [9]. Comparative transcriptomic analysis of prion disease responses between mice and rats identified shared neuroinflammatory responses, however, at the level of individual genes homologous responses were not conserved and many genes deregulated by prion infection in mice were unaffected in rats [31]. The present study provides a comprehensive profile of gene-expression changes in response to CWD in the brain of CWD-infected deer. We evaluated differences between clinically affected and healthy white-tailed deer using bulk RNA-seq, assessing changes in the neural transcriptome of infected animals to define the molecular pathology of this universally fatal disease.

Consistent with observations in rodent prion diseases [31, 32] and neurodegenerative diseases with comparable pathology in humans [33], our data confirm the up-regulation of neuroinflammatory responses observed in many prion diseases. Several ontologies related to inflammation were enriched among the upregulated genes, with multiple genes related to innate and complement immune activation showing upregulation in the diseased deer. Prion diseases are characterized by the early onset of gliosis in the brain of the infected animal with microglial and astrocyte proliferation occurring simultaneously with the accumulation of prion aggregates [34], and often prior to neuronal loss, spongiform change, and clinical signs. The expression levels of genes involved in microglia activation were higher in CWD+ animals. Microglia are the resident macrophages of the CNS, playing a key role in the maintenance of homeostasis via surveillance and phagocytosis in the brain. Survival and proliferation of microglia relies on signaling via the colony stimulating factor 1 receptor (CSF1R) [35], whose gene was upregulated in the CWD+ deer. In mouse prion disease, CSF1R increases microglial activation and promotes neurodegeneration [36]. CSF1R upregulation by CWD in white-tailed deer is indicative of microglial activation. The upregulation of the CXCR4 gene in the CWD+ animals supports microglial activation. CXCR4 is a chemokine receptor with broad modulatory functions in immune system regulation, that also regulates apoptosis and neuronal guidance through astroglial signaling and microglial activation [37]. A recent meta-analysis found that upregulation of this gene was a common pathology of multiple human neurodegenerative diseases, perhaps indicative of conserved processes underpinning these and prion diseases [37, 38].

Further evidence of microglia proliferation was seen in upregulation of complement receptor genes. The complement system is an essential component of innate immunity, important in protecting from infection and repairing tissue damage [39]. Activation of complement also contributes to the neuroinflammation and tissue damage accompanying traumatic brain injury [40], and a role(s) for complement has been demonstrated in human cases of prion [41], Alzheimer’s, and Huntington’s diseases [42]. Complement activation fragments, C3a and C5a, interact with cellular receptors to recruit and activate microglia and clear pathogens [43]. Genes encoding both known C5a receptors (*C5AR1* and *C5AR2*) were upregulated in our dataset, indicating neural complement activation accompanies CWD in deer.

Both protective (M1) and neurotoxic (M2) phenotypes of microglial activation in prion diseases have been proposed [44]. Inhibiting microglial activation increased survival time and delayed the onset of clinical symptoms in rodent prion diseases [36], but complete absence of microglia accelerated death in prion-infected mice [45]. The role of microglial activation in prion disease changes with advancing illness, from phagocytosing infectious prions during early infection, to the promotion of inflammation leading to further degeneration in clinical disease. A strong relationship between the expression of immune-related genes and CWD diagnosis has been reported in the retropharyngeal lymph nodes of white-tailed deer [9], and in Rocky Mountain elk [8].

Dysregulation of calcium binding / signaling pathways is thought to be contributory to prion-induced cell death, and was associated with CWD in deer and elk [8, 9]. The three most upregulated genes in our dataset encoded the A8, A9, & A12 family members of the *S100* protein family (Additional file 2). This multifunctional family of calcium-binding proteins have been implicated in Alzheimer’s disease (AD) pathogenesis, and are upregulated in cases of traumatic brain injury, natural aging, and neuronal damage [46]. Elevated levels of S100 proteins can be used as a clinical biomarker for CJD diagnosis in humans [47], and these proteins were also enriched in a murine model of scrapie [48], indicating a conserved role in TSEs across species. Many neurodegenerative diseases are accompanied by a similar pattern of chronic neuroinflammation, contributing to tissue degeneration. S100A8 and S100A9 heterodimerize to form calprotectin in vivo, and their expression is modulated by pro-inflammatory cytokine expression as part of the innate immune response to protein aggregation in the brain. This heterodimer is upregulated in microglial cells that collocate with amyloid plaques in the brain during AD. S100A9 in particular is implicated in neurodegeneration, with its intranasal application inducing cellular stresses in the brain as well as impairing learning ability [9].

While prions are accepted as the causative agents of TSEs, there is a hypothesis that *Spiroplasma* spp. in lymphatic or neural tissues may be the etiological agent responsible for the transmissible spongiform encephalopathies [29]. As such we took a metagenomic approach to investigate if there is a microbial community in the brains of deer and in particular, one that is specific to CWD+ deer. Of the ~ 46 million RNA-seq reads generated per sample, only ~ 0.04% of them could be assigned to a bacterium by Kraken2. The most likely explanations for the mapping of reads from a deer brain sample to bacterial genomes are contamination of the sample by commensal bacteria, chance misassignment of unannotated deer transcripts to any of the bacterial genomes deposited in NCBI, or contamination of molecular biology reagents used to prepare the RNAseq libraries. We see examples of all of these in the RNAseq bacterial assignments. Both *Staphylococcus haemolyticus* and *Escherichia coli* are common flora of mammals. The presence of reads from *Oceanobacillus iheyensis* is extremely unlikely as this bacterium is a halotolerant bacillus from deep sea sediment [49]. Contamination of RNAseq datasets by *Streptomyces* reads has previously been described [50] and *Streptomyces lavendulae,* in particular, is used for biopharmaceutical production. While we did see evidence of bacterial transcripts in the RNASeq data, there was no bacterial signal that correlated with disease status. Less than 0.00008% of the reads mapped to genes from *Spiroplasma* species, about 38 reads. These data do not corroborate the hypothesized role of *Spiroplasma* or any other bacteria in the etiology of CWD and support previous data refuting the hypothesis [51, 52].

RNAseq analysis of the preclinical case, NBSK23, was indeterminate and it did not cluster with the clinical samples. While the deer was positive for CWD in both the retropharyngeal lymph node and obex, the abundance of PrPCWD in the whole brain sample was substantially lower than in the clinical deer samples. As prion infections all have a relatively long preclinical period when infectious prions accumulate in the absence of clinical signs, it is prion accumulation in this deer was insufficient to elicit measurable neurodegenerative and neuroinflammatory changes.

This study presents the first comprehensive transcriptome analysis of CWD in the brain tissue CWD-positive cervids. In our studies, we observed inter-animal variation between CWD+ animals in both RNASeq and qPCR data, indicative of a non-uniform response to disease. Our deer samples were collected from different cohorts of wild deer and included experimentally infected animals as well as hunter-harvested and captive deer. This stands in contrast to the majority of data in the literature concerning neural transcriptional responses to prion diseases that use cohorts of inbred mice. One deer samples, 1285, had a prion protein polymorphism (wt/96) known to slow CWD progression. Deer with the wt/96 genotype survived 38% longer than deer carrying two wild-type alleles [12]. Our sample size was limited by the difficulty of carrying out long-term experiments in captive cervids under biosecure conditions.

## Summary & Conclusions

This study examined the transcriptional response of the brain to CWD infection in white-tailed deer. We demonstrated the large-scale neuroinflammation that accompanies CWD infection is underpinned by activation of both microglia and the complement system. The gene expression changes highlight the role of innate immunity in prion infection providing for disease biomarkers and for potential targets for therapeutic agents. Finally, we mined our data to address the speculative hypothesis that specific bacteria are the etiological agent of CWD, but found no evidence to support this hypothesis.

## Supplementary Information

**Additional file 1: Supplementary Table 1**. Primers and annealing temperature for qRT-PCR studies. **Supplementary Figure 1**. Cluster dendrograms of gene expression profiles across sequencing batch and disease phenotype. Dendrogram prepared using log-transformed counts per-million. The clustering analysis considered all genes in the data, not just those affected by treatment. **Supplementary Figure 2**. Heatmap depicting the VST normalized abundances of the 40 predominant bacterial species detected in neural deer tissue. Species are ranked by abundance, while columns (samples) are clustered by similarity (Ward). Heatmap prepared in R using the pheatmap package. **Supplementary Figure 3**. Multiple exposures of Fig. 1.

**Additional file 2. **Differentially expressed gene lists.

## Data Availability

The datasets generated and/or analyzed are available in the [National Center for Biotechnology Information BioProject] repository, accession number PRJNA756812, (www.ncbi.nlm.nih.gov/bioproject/PRJNA756812).
