# Supplementary material for: Neural transcriptomic signature of chronic wasting disease in white-tailed deer
Source: BMC Genomics. 2022 Jan 21;23:69. doi: 10.1186/s12864-022-08306-0 (PMC8783489; doi:10.1186/s12864-022-08306-0)

**Neural transcriptomic signature of chronic wasting disease in white-tailed deer.**

Eóin O'Hara^1^, Allen Herbst^1^, Arun Kommadath^2^, Judd M. Aiken^1^, Debbie McKenzie^3^, Negin Goodarzi^4^, Pamela Skinner^4^, Paul Stothard^1^

**Supplementary Table 1. Primers and annealing temperature for qRT-PCR studies**

| **Gene symbol** | **Gene ID** | **Forward primer** | **Tm** | **Reverse Primer** | **Tm** | **product size** | **Intron?** |
| --- | --- | --- | --- | --- | --- | --- | --- |
| CD14 | 110129335 | ACGACGACGATTTCCGCTGT | 62.47 | CCACCTCGACGGCAACCATA | 62.23 | 90 | No |
| TREM2 | 110124385 | TGCATCTGTCCTCCACAAGGG | 62.07 | ACCGTGGTGTTGTGCGCT | 62.85 | 98 | No |
| ABCC3 | 110150921 | TGCCAACTTCCTCCGCAACT | 62.34 | TTTGTCCTCCAAGGCTGGCT | 61.72 | 82 | No |
| ITGB3 | 110131336 | AGCTCCTGCCAGCAATGTCT | 61.85 | CTCCTTCAGGTTACAGCGGGG | 62.45 | 96 | Yes |
| CD163 | 110139304 | CCCTGTGGGATTGTCCTGCC | 62.84 | GAGTGACCTGCGGCCTTTAGT | 62.38 | 121 | Yes |
| GPNMB | 110126680 | ATGCCAGCGGCAACATAGTC | 61.1 | TGTCCACGCTGTCCAGTTGT | 61.9 | 92 | No |
| CD68 | 110121810 | ACGGACAGCTGAGTTTCGGATT | 62.24 | TGAGAATGTCCACTGCACTGCC | 63.08 | 116 | Yes |
| ITGB2 | 110125845 | CTGACACACCTGAGCGACCT | 61.81 | GACTGTCGTGGTGGCACTCT | 62.09 | 108 | Yes |
| EMP1 | 110131254 | GGTCCACATTGCCACCGTC | 61.33 | CGGAATCTGACACCACCCAGA | 61.78 | 71 | Yes |
| LCP1 | 110125076 | AACCAGGACATCGACTGGGG | 61.55 | CCAGGGCATCTGATAAGTCACTGT | 62.2 | 130 | Yes |
| C5AR1 | 110139889 | AGCCCTCGGGAGCCTTTGA | 63.23 | ATGGTGTTGGGGTTCGAGGTC | 62.33 | 119 | Yes |
| MYOC | 110123806 | GCCATCAGGCTCCAGGGAAG | 62.62 | ATTTGGGAAGCAGGAACCTCTGT | 62.11 | 96 | Yes |
| SCNN1D | 110124977 | ACGGAGACCACCATCGACATT | 61.51 | ACACTGCCTGTGCTGTCCAT | 62.06 | 86 | Yes |
| RGS1 | 110137654 | ACATCTGGAATCTGGAATGAAGCCT | 62.11 | CTCCAGGGATTCAGACCACTGC | 62.64 | 86 | Yes |
| BActin | [110125611](https://www.ncbi.nlm.nih.gov/gene/110125611) | CCATGTACCCTGGCATCGCA | 62.61 | CAGGGGGCGCGATGATCTTTAT | 62.83 | 90 | Yes |


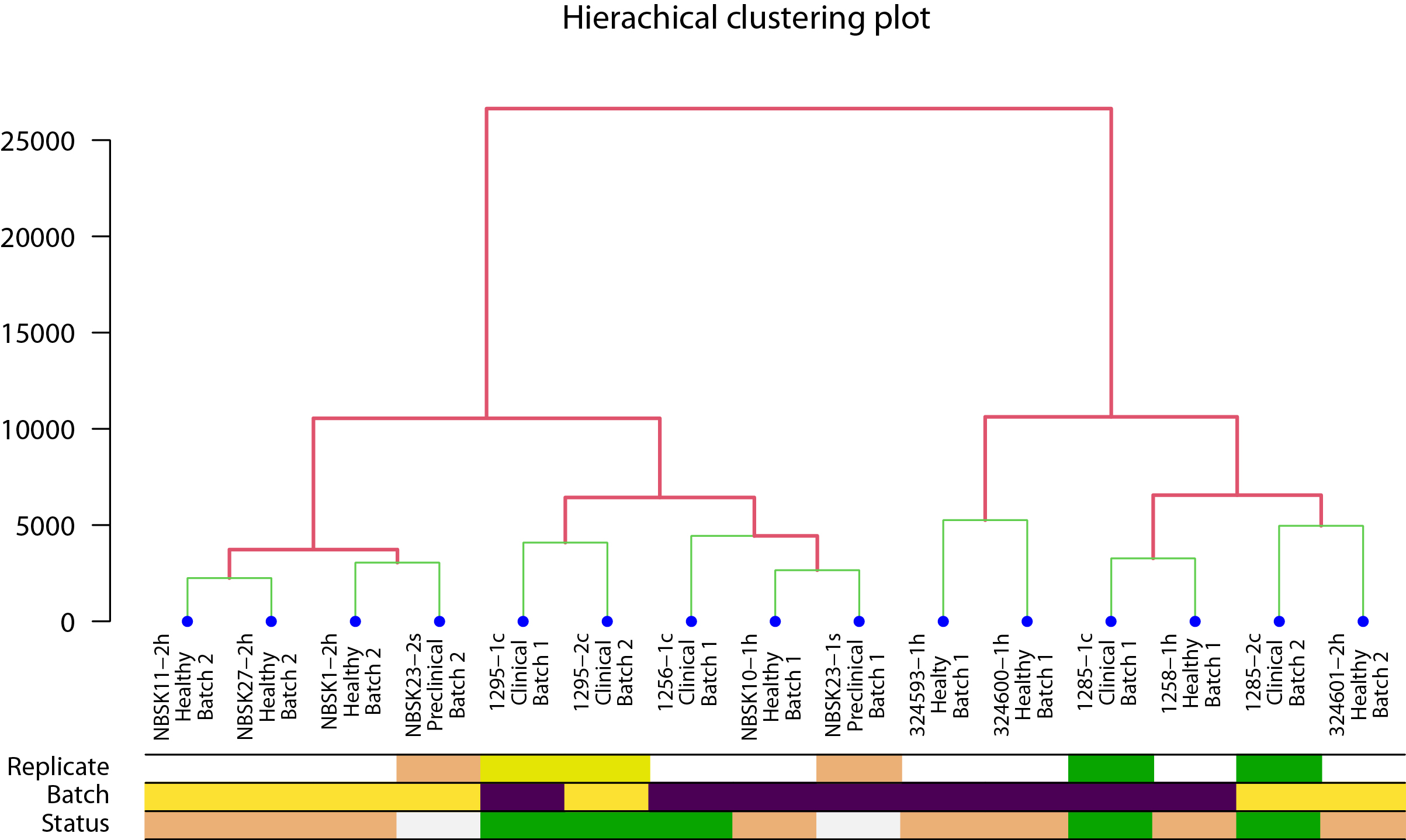


**Supplementary Figure 1.** Cluster dendrograms of gene expression profiles across sequencing batch and disease phenotype. Dendrogram prepared using log-transformed counts per-million. The clustering analysis considered all genes in the data, not just those affected by treatment.


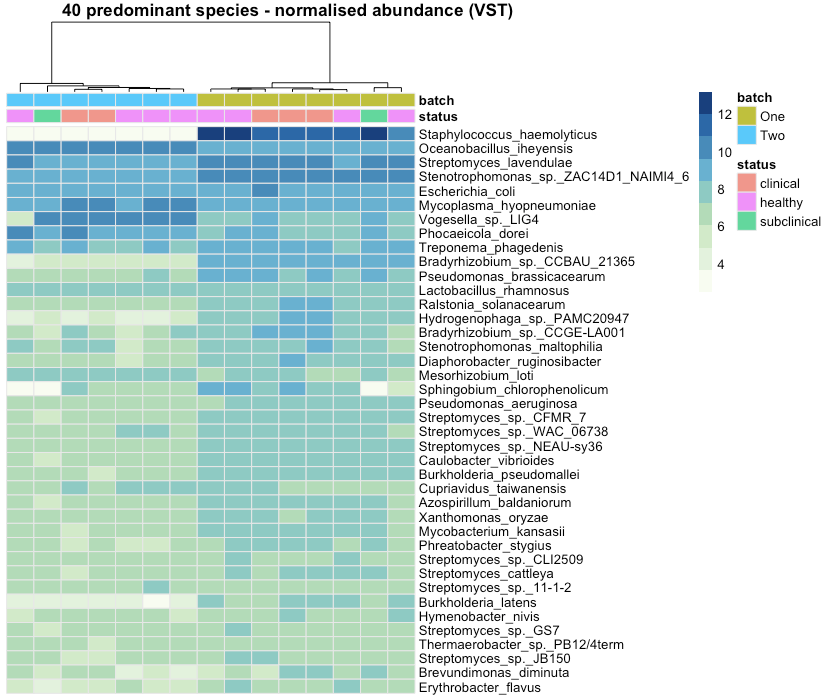


**Supplementary Figure 2.** Heatmap depicting the VST normalized abundances of the 40 predominant bacterial species detected in neural deer tissue. Species are ranked by abundance, while columns (samples) are clustered by similarity (Ward). Heatmap prepared in R using the pheatmap package.

**Supplementary Figure 3.** Multiple exposures of figure 1.


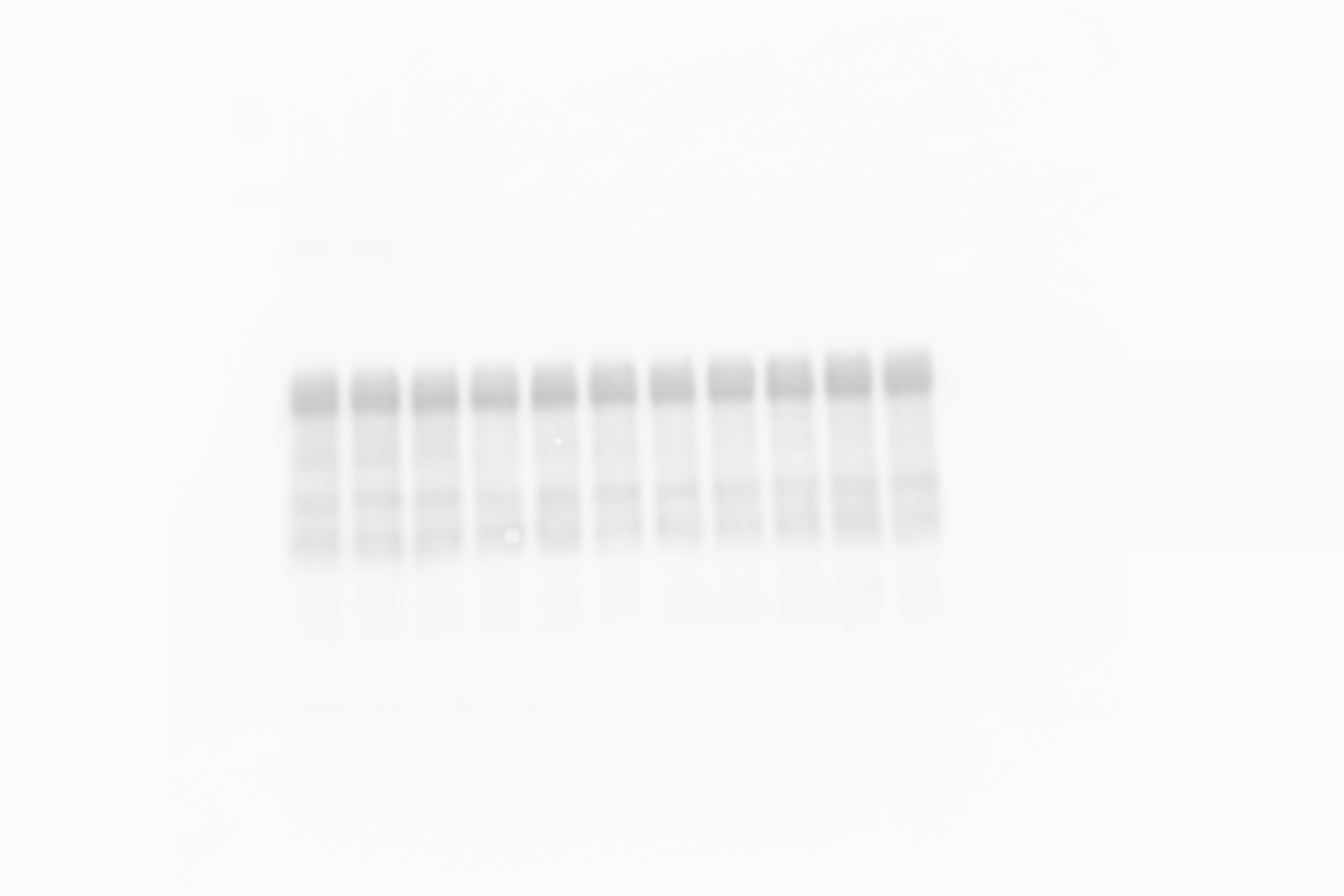

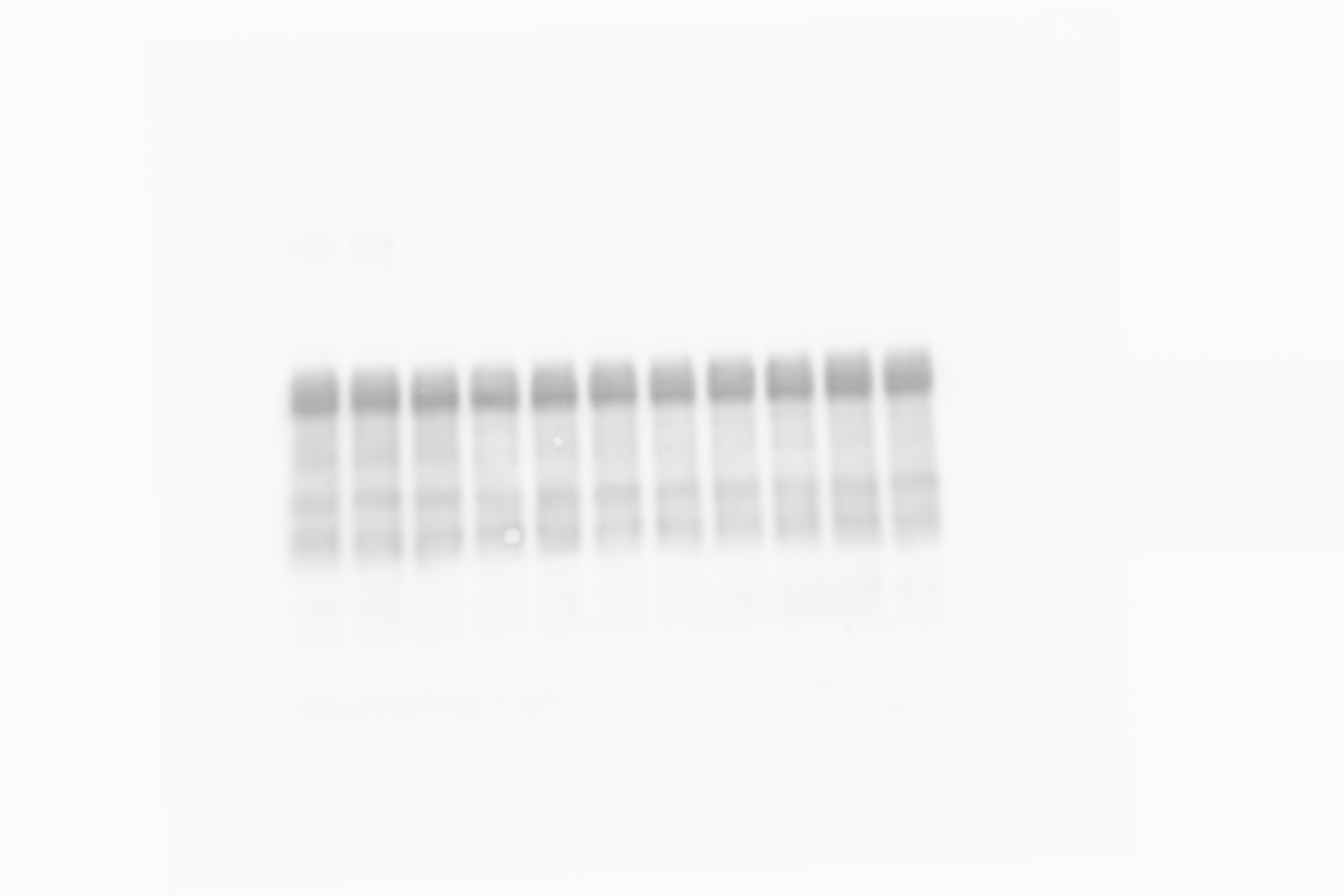

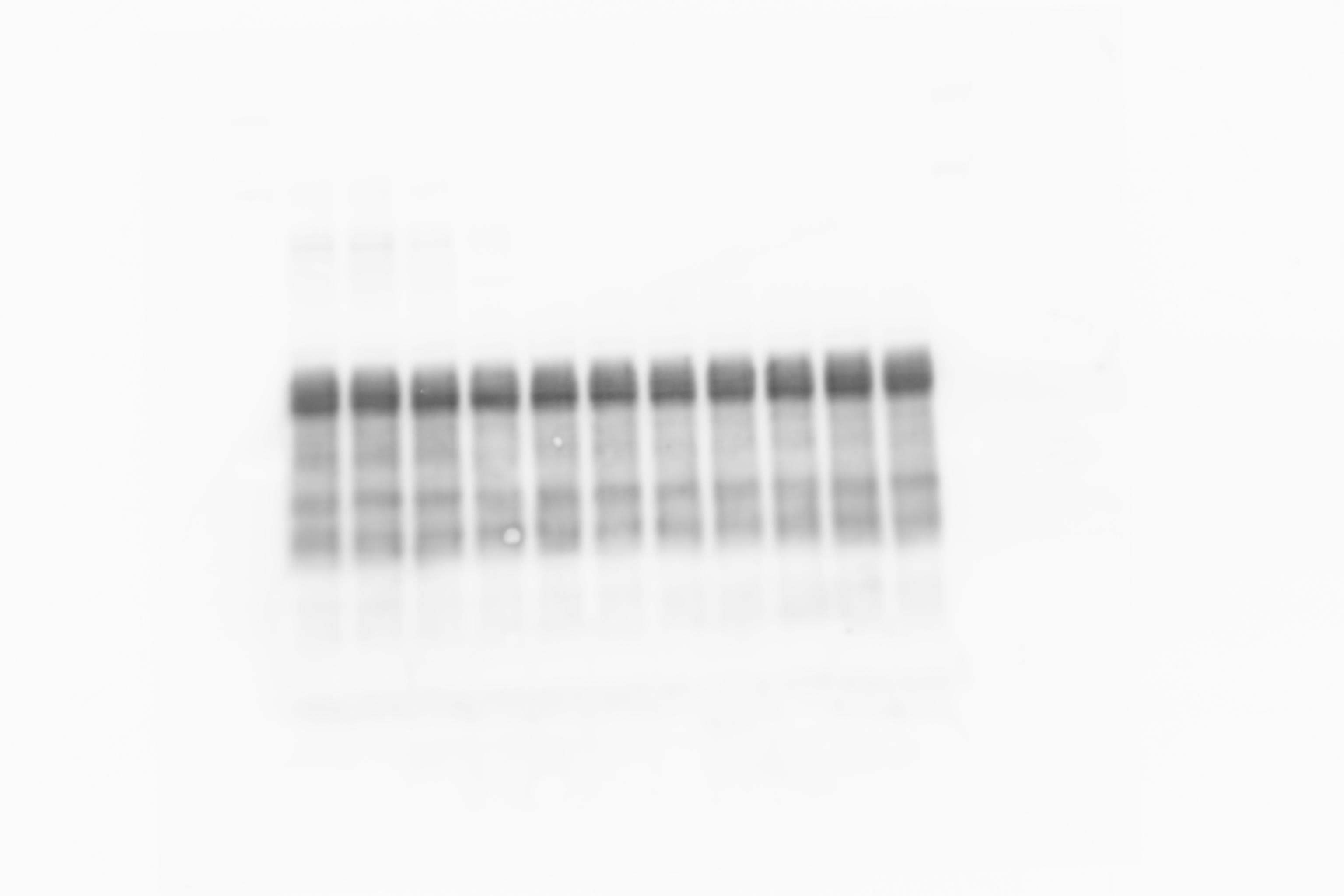

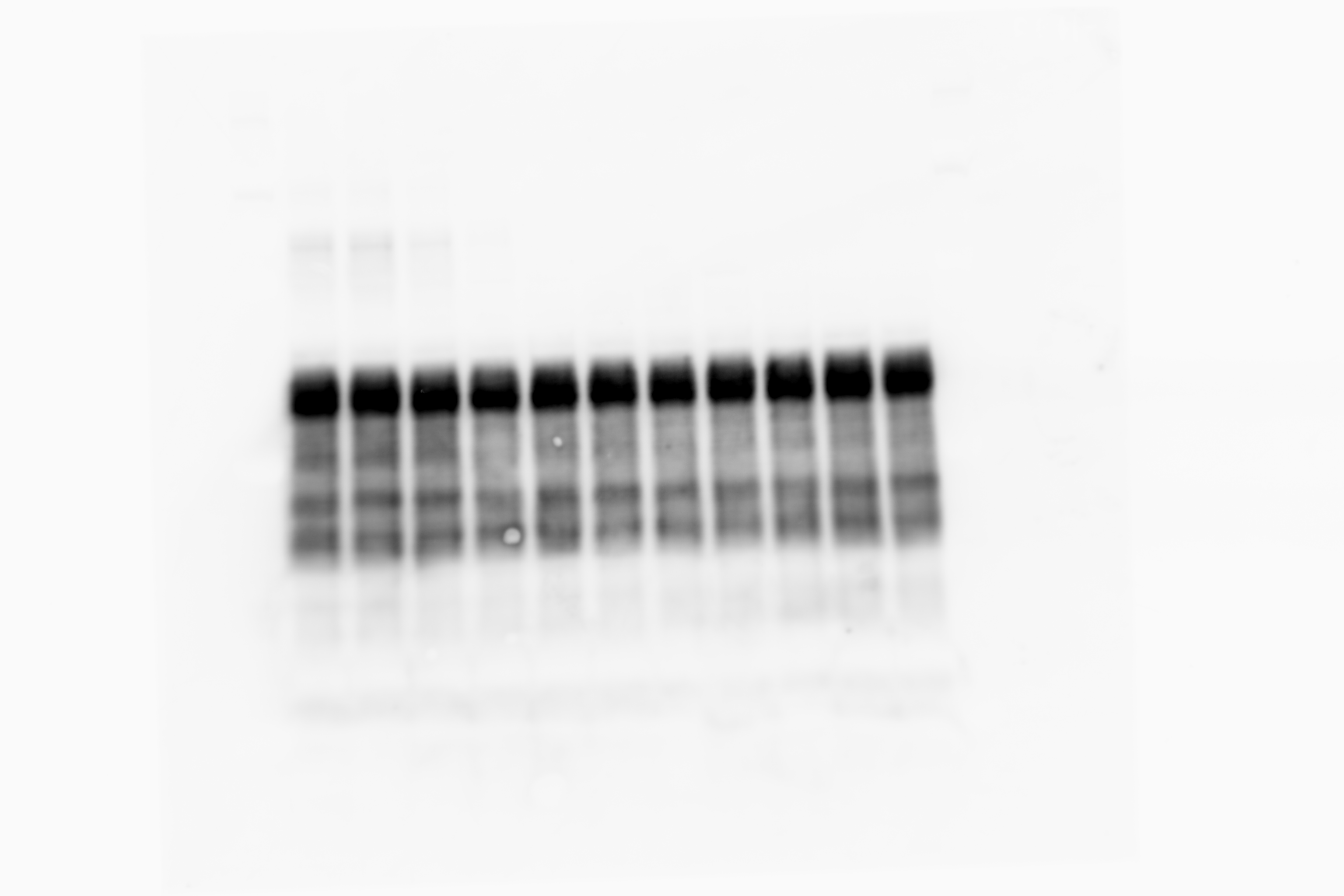

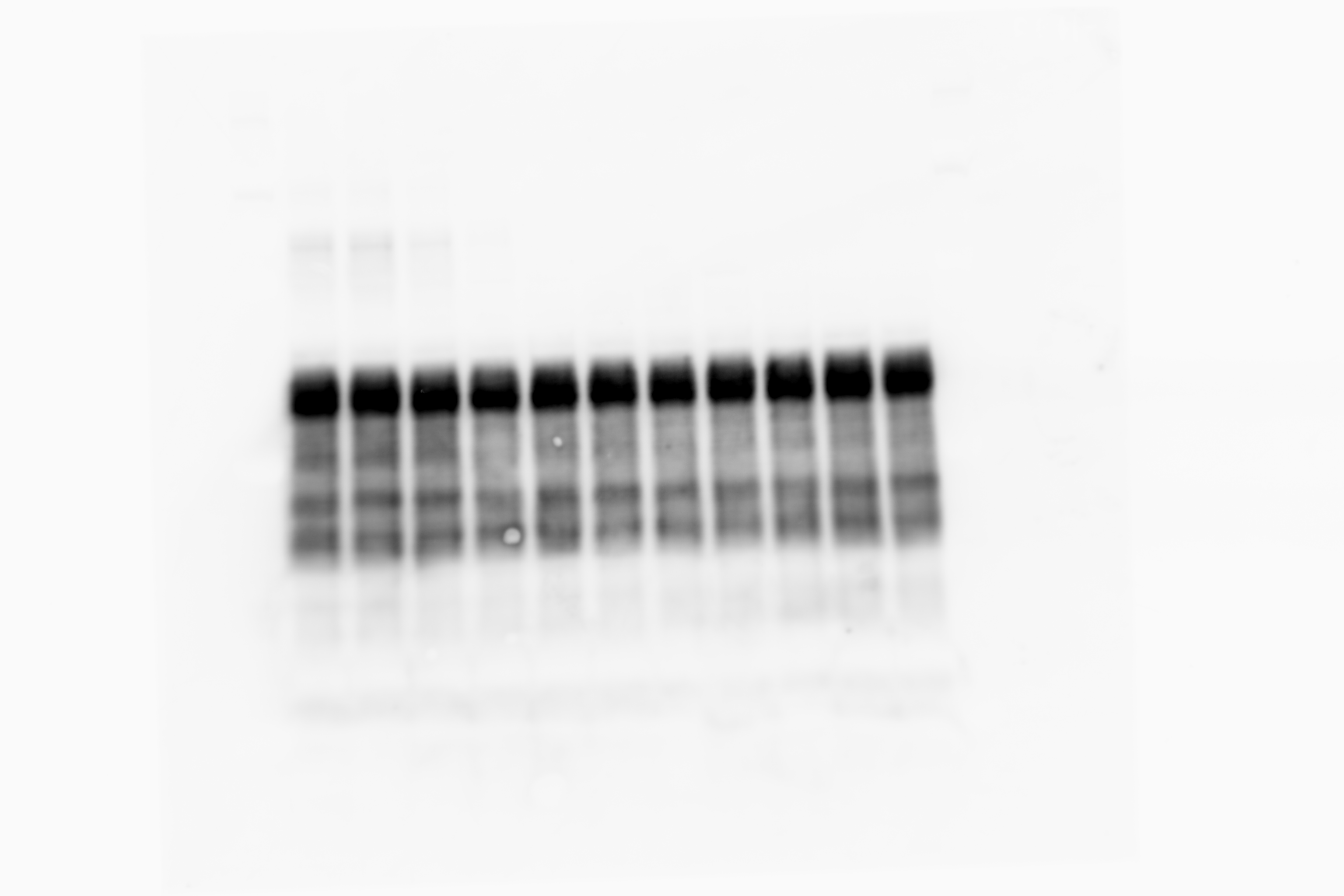

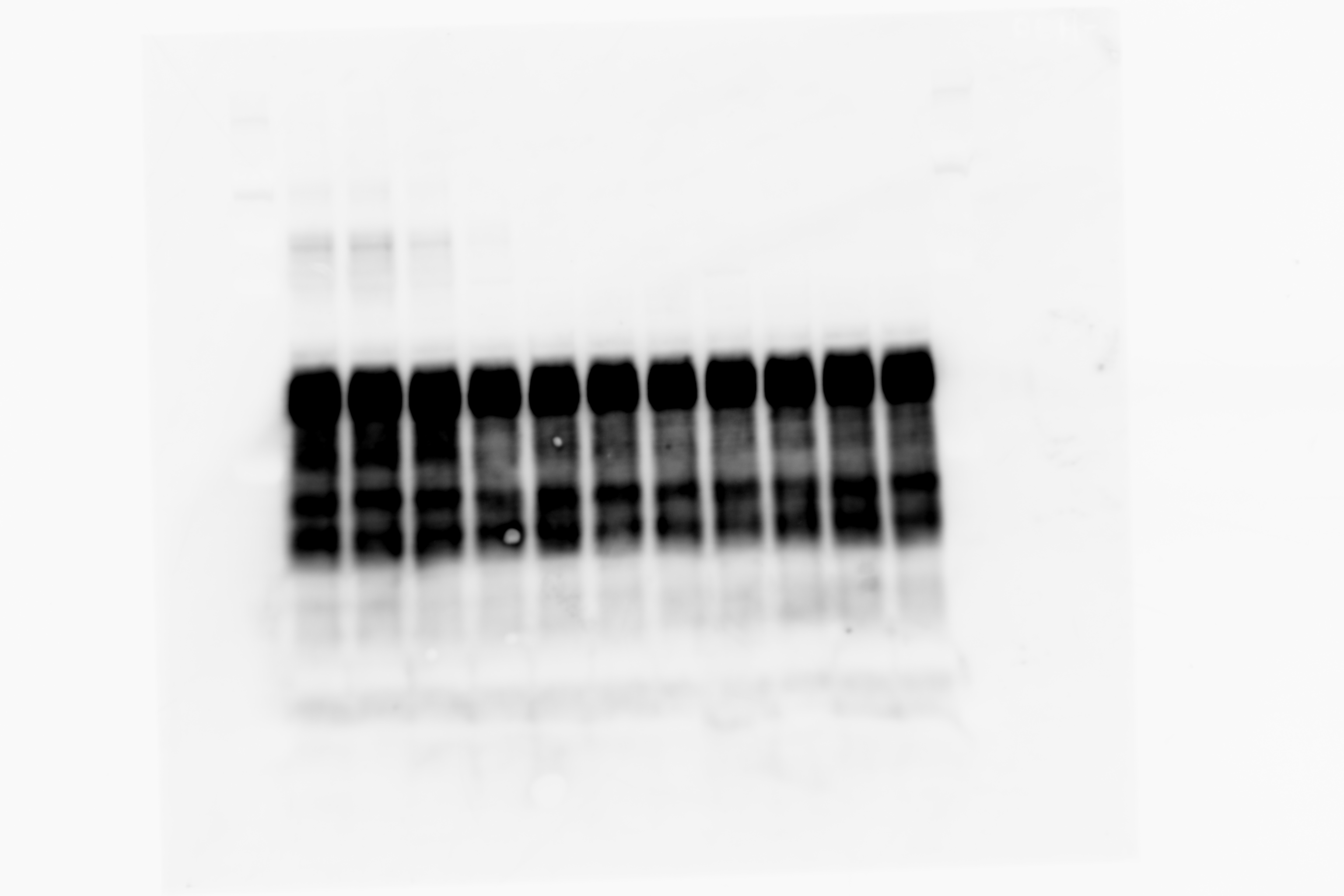

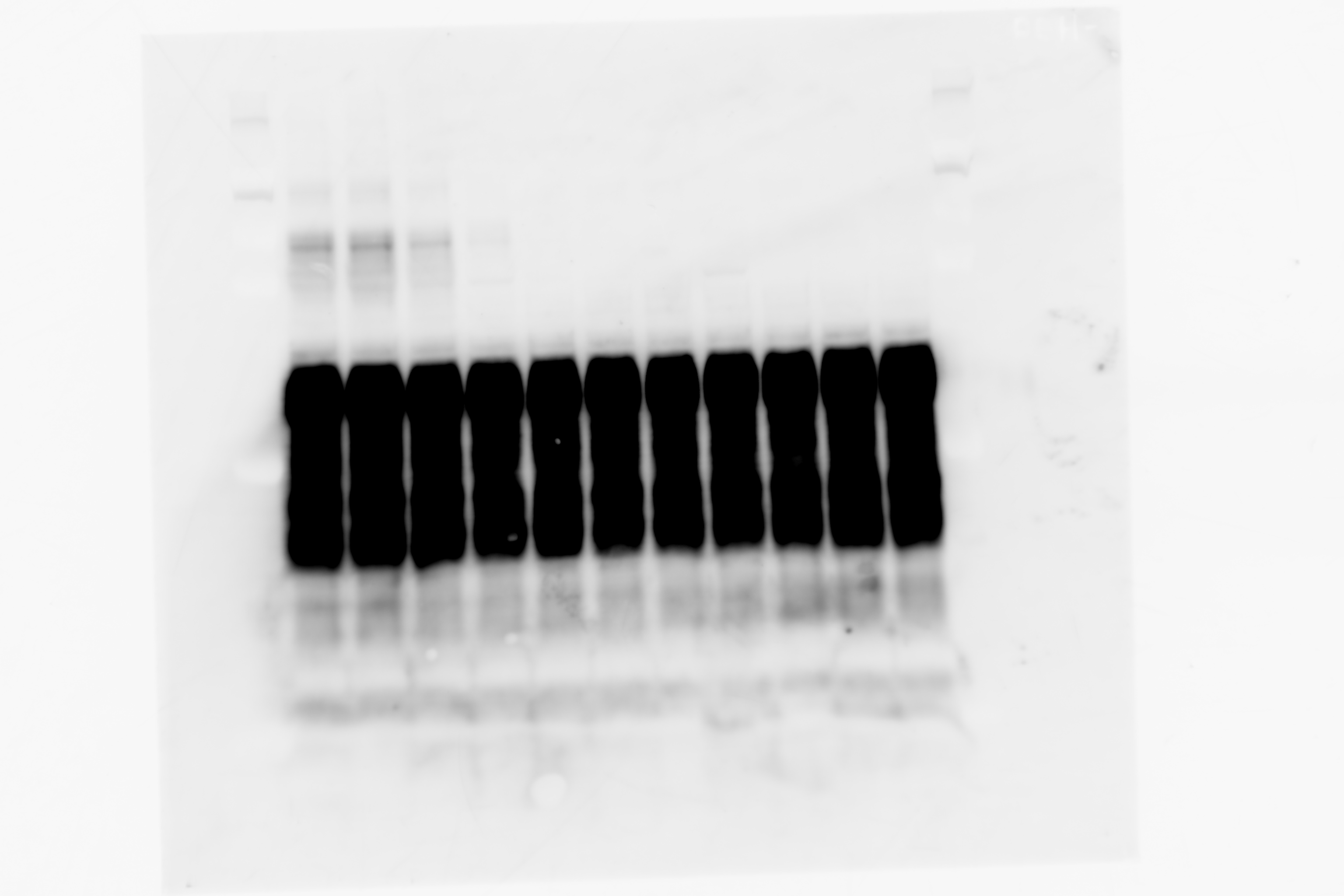

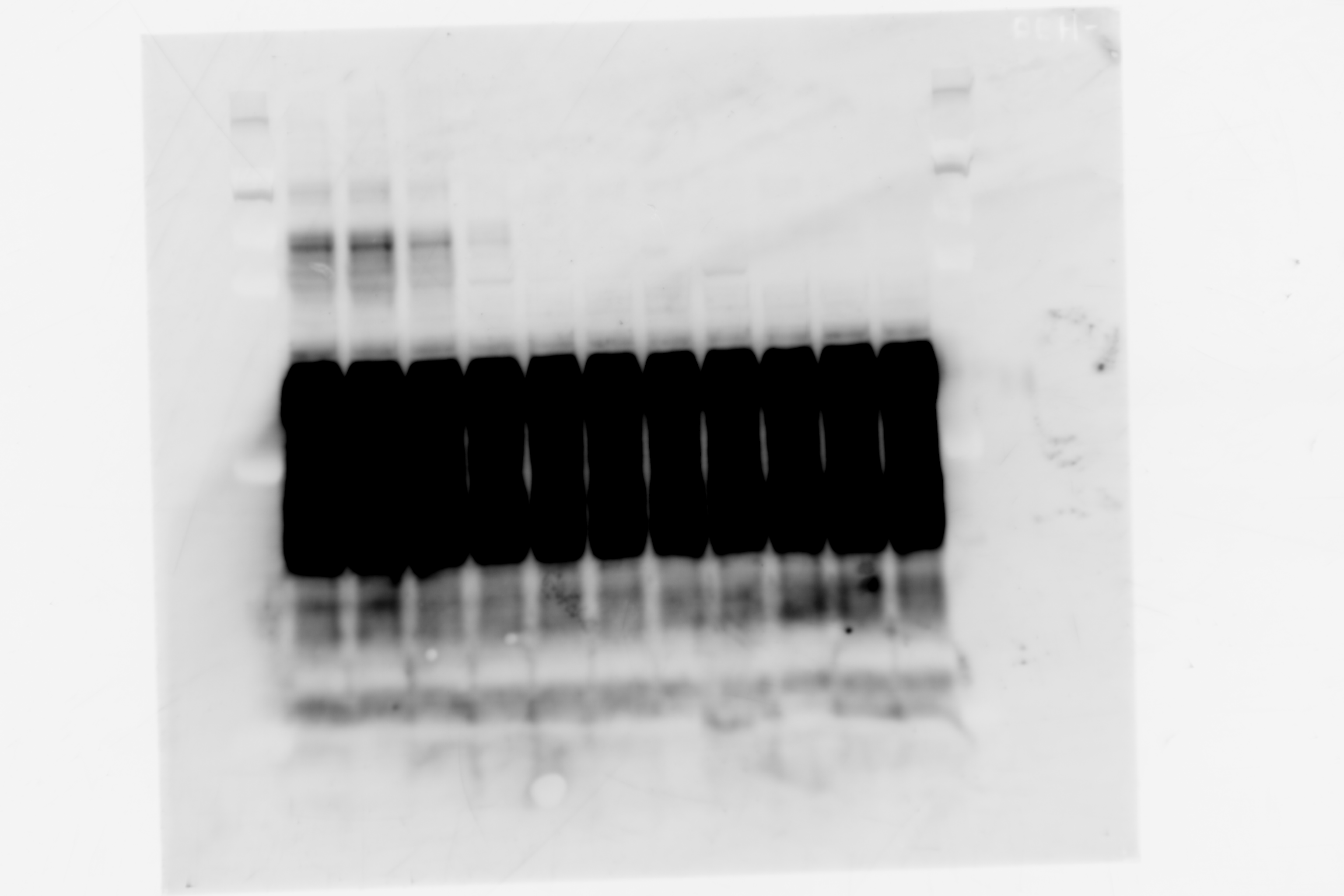

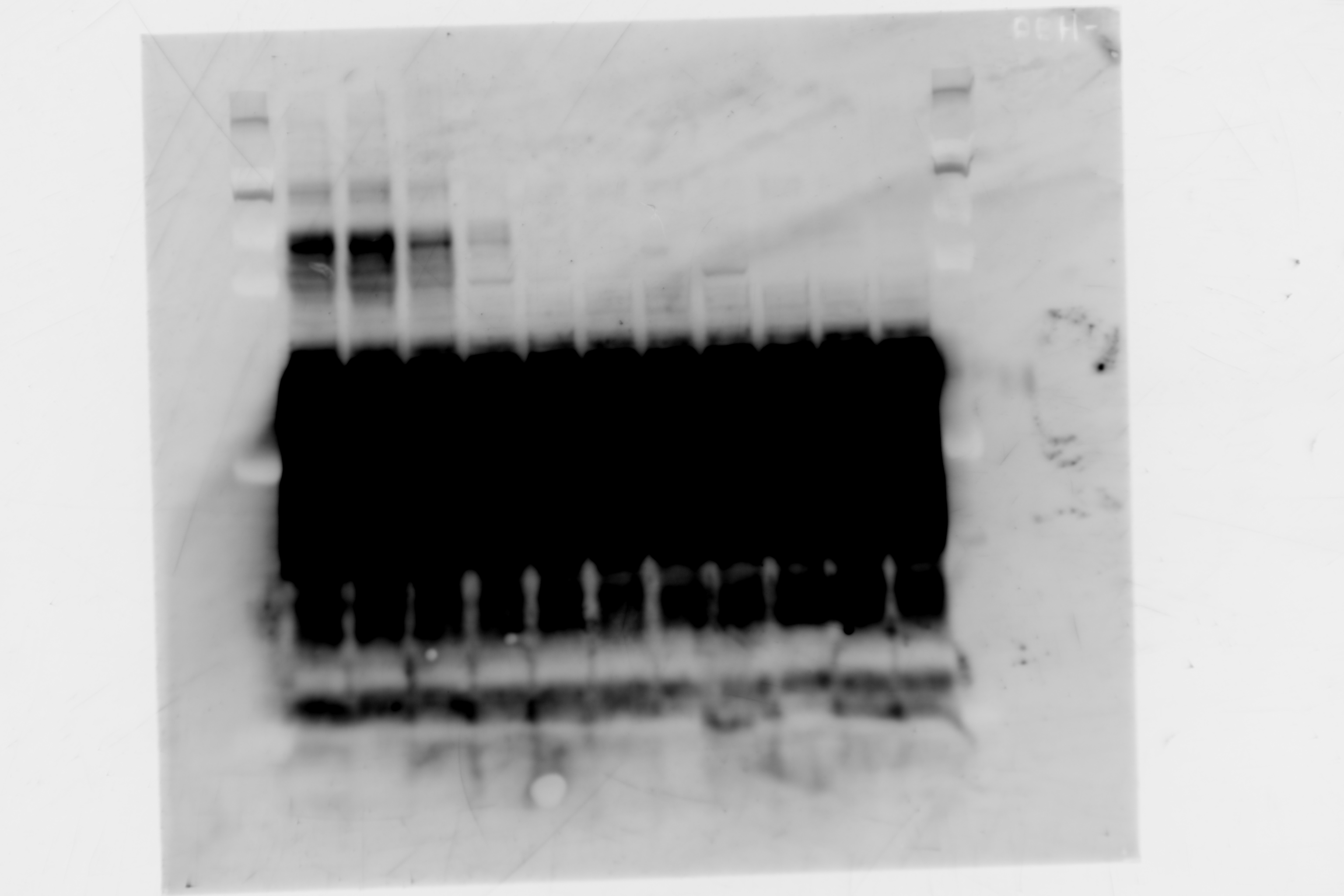

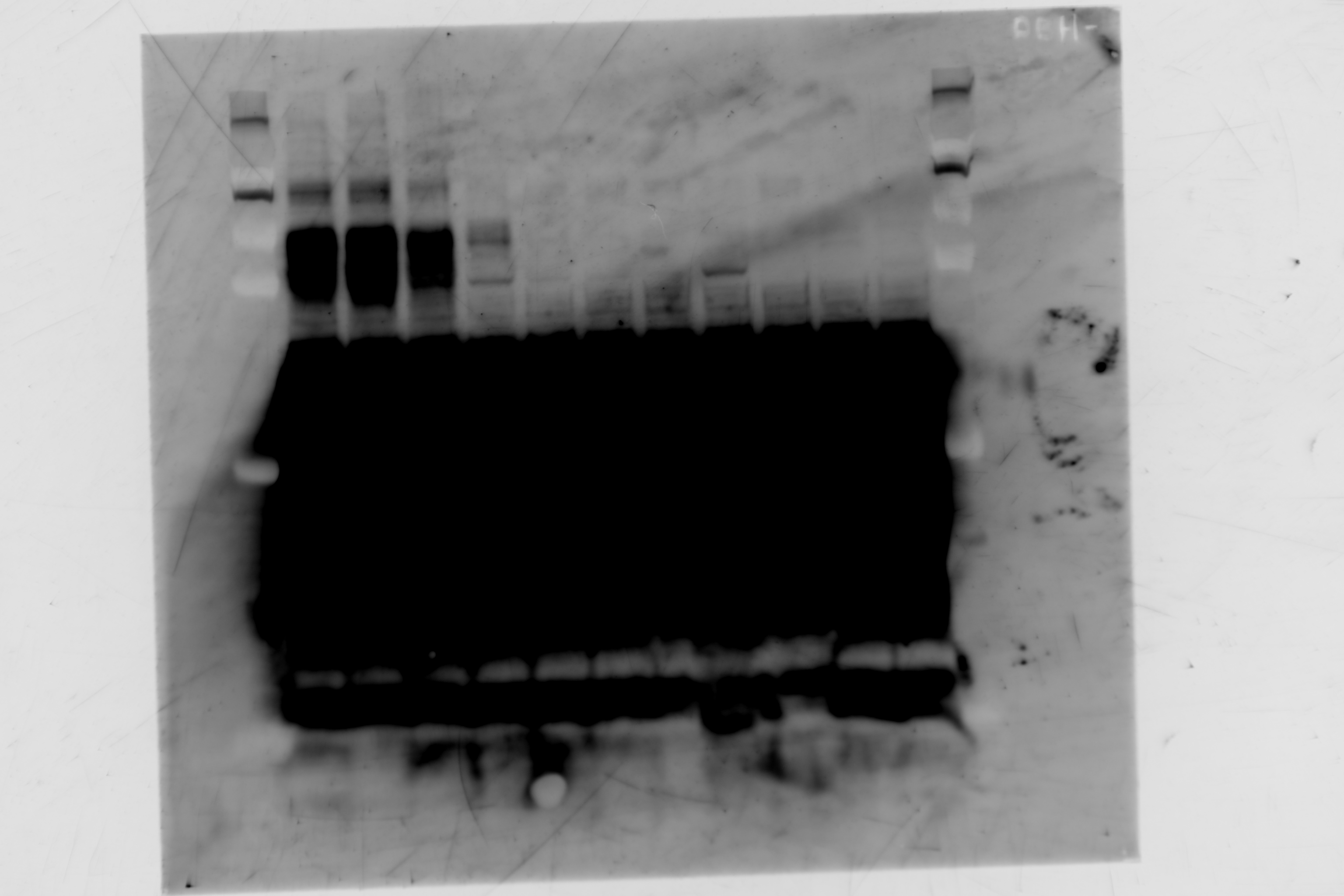

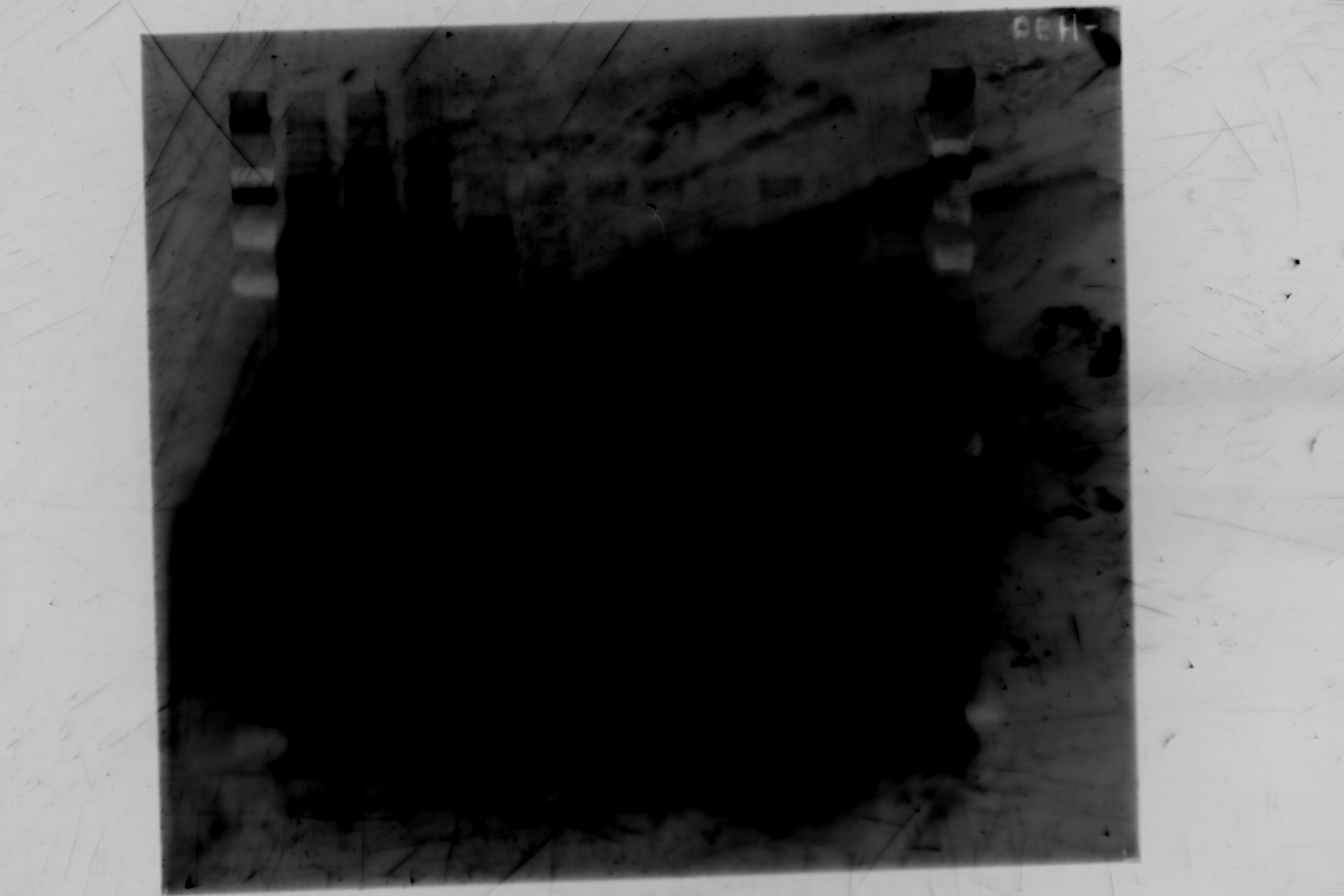

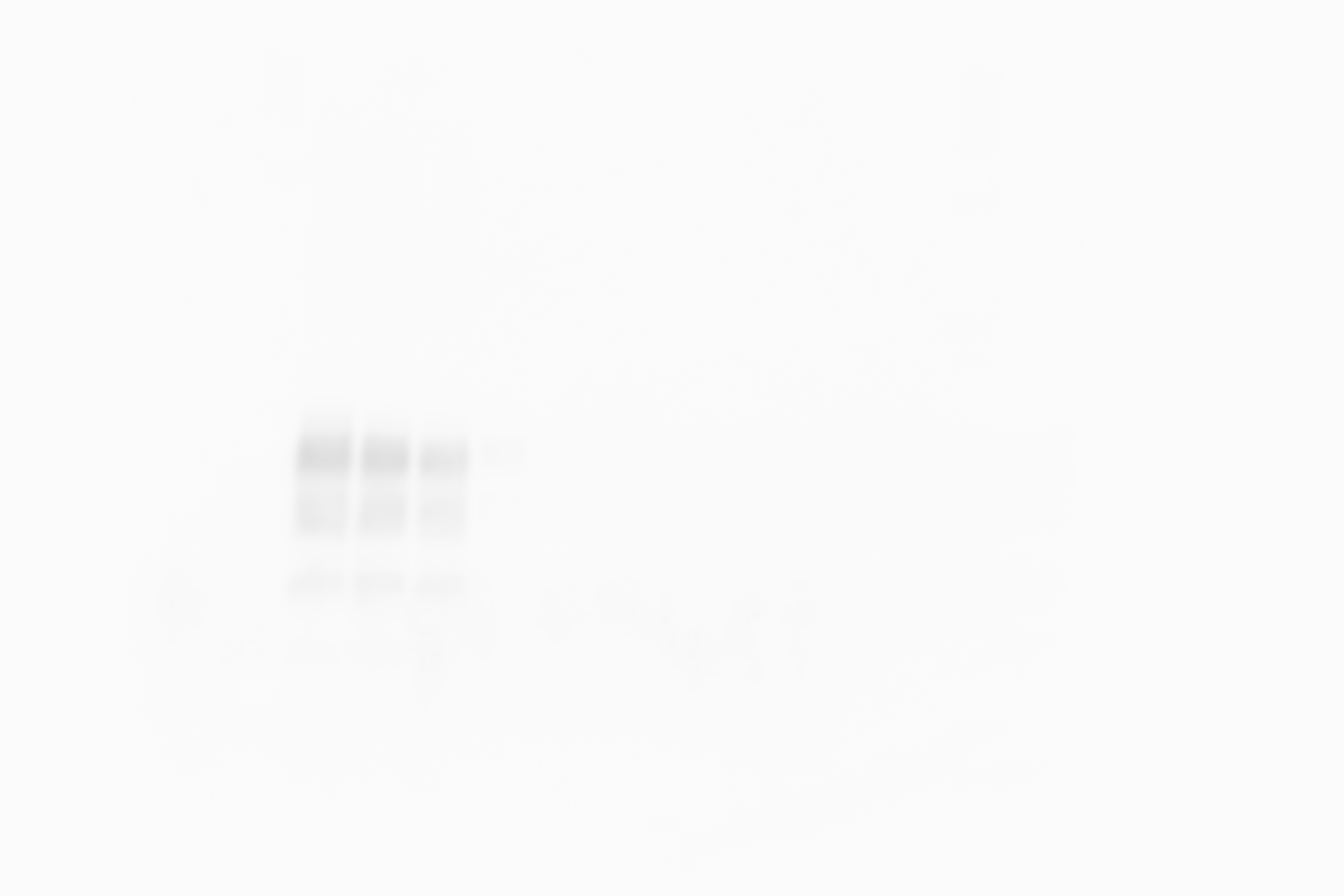

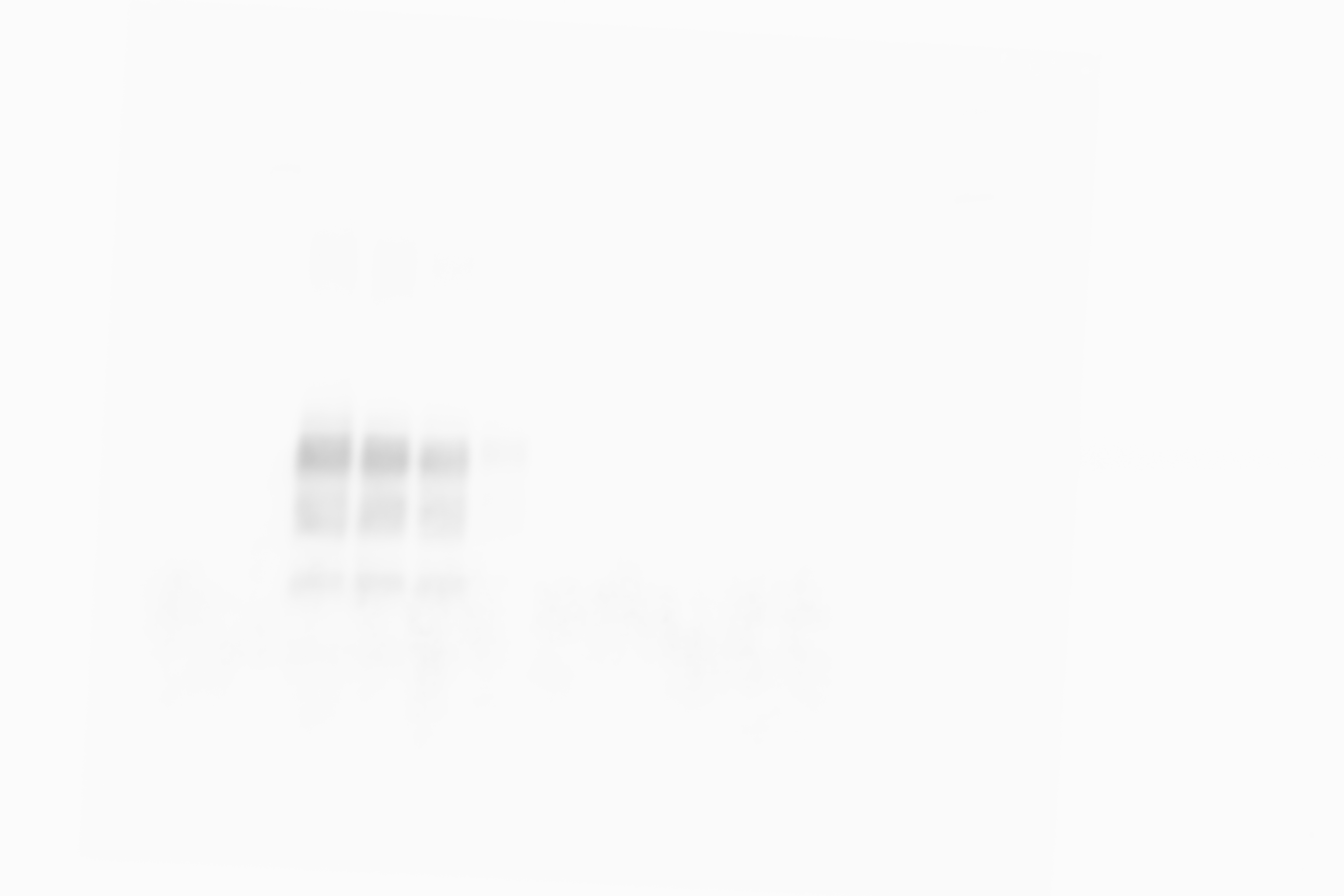

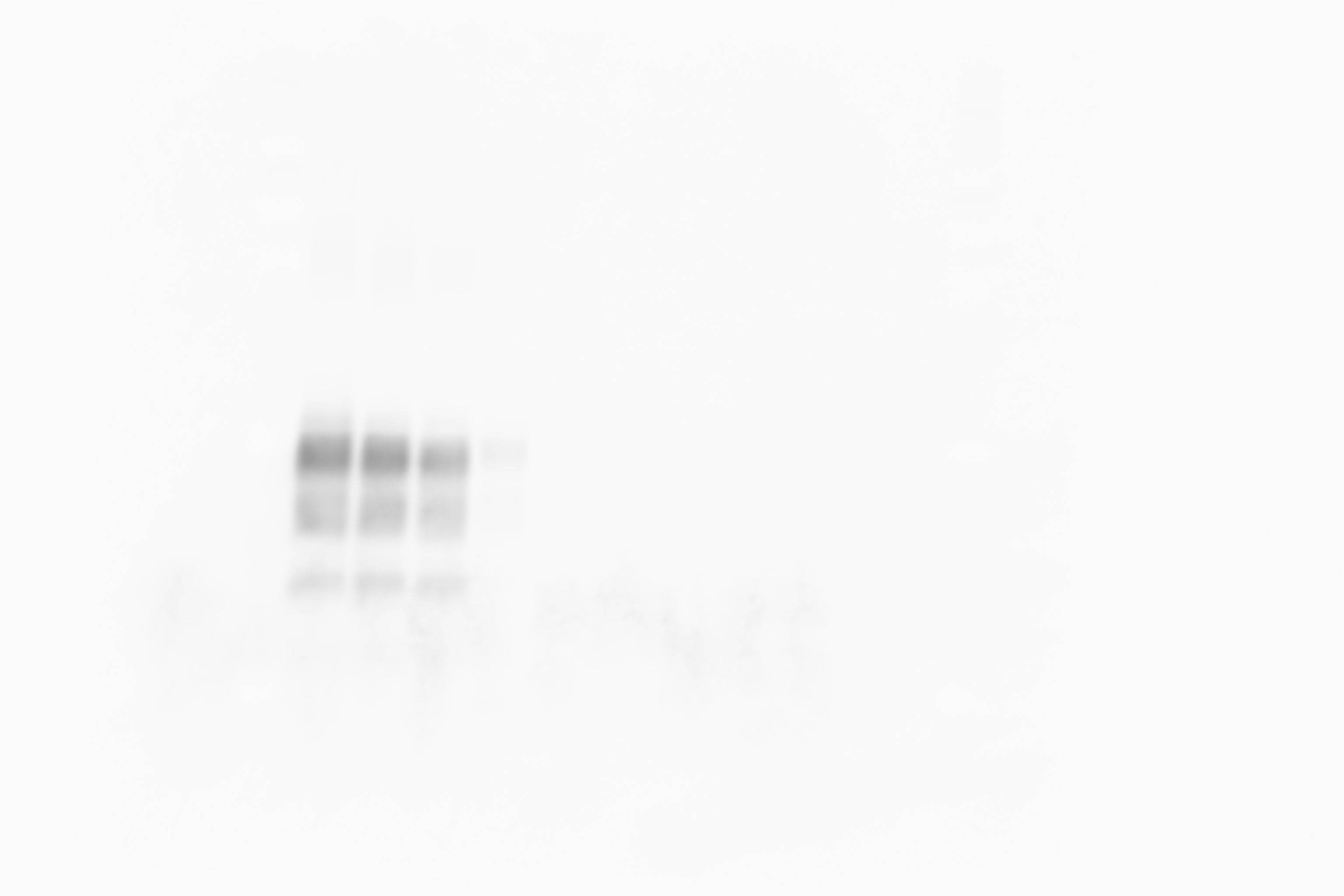

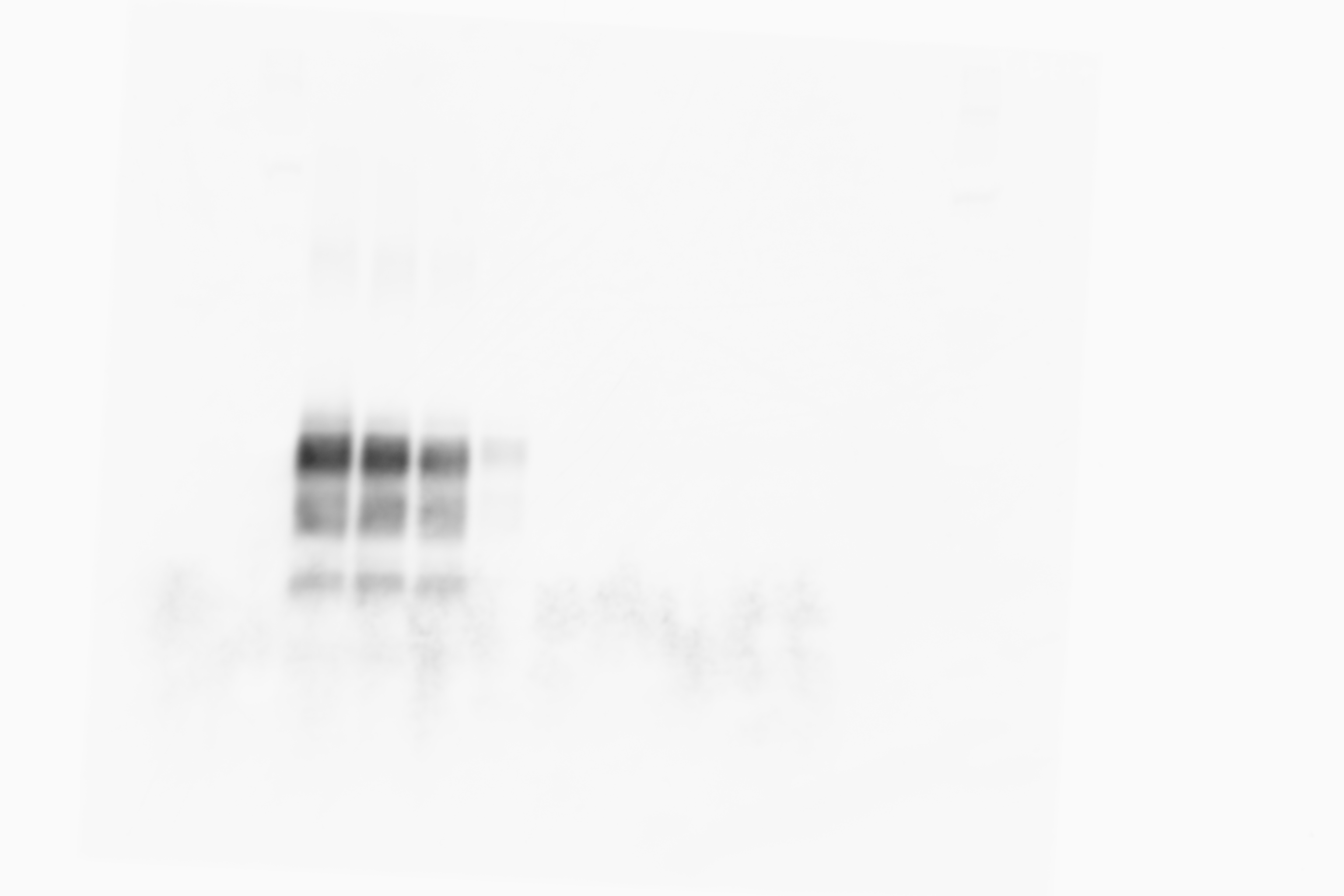

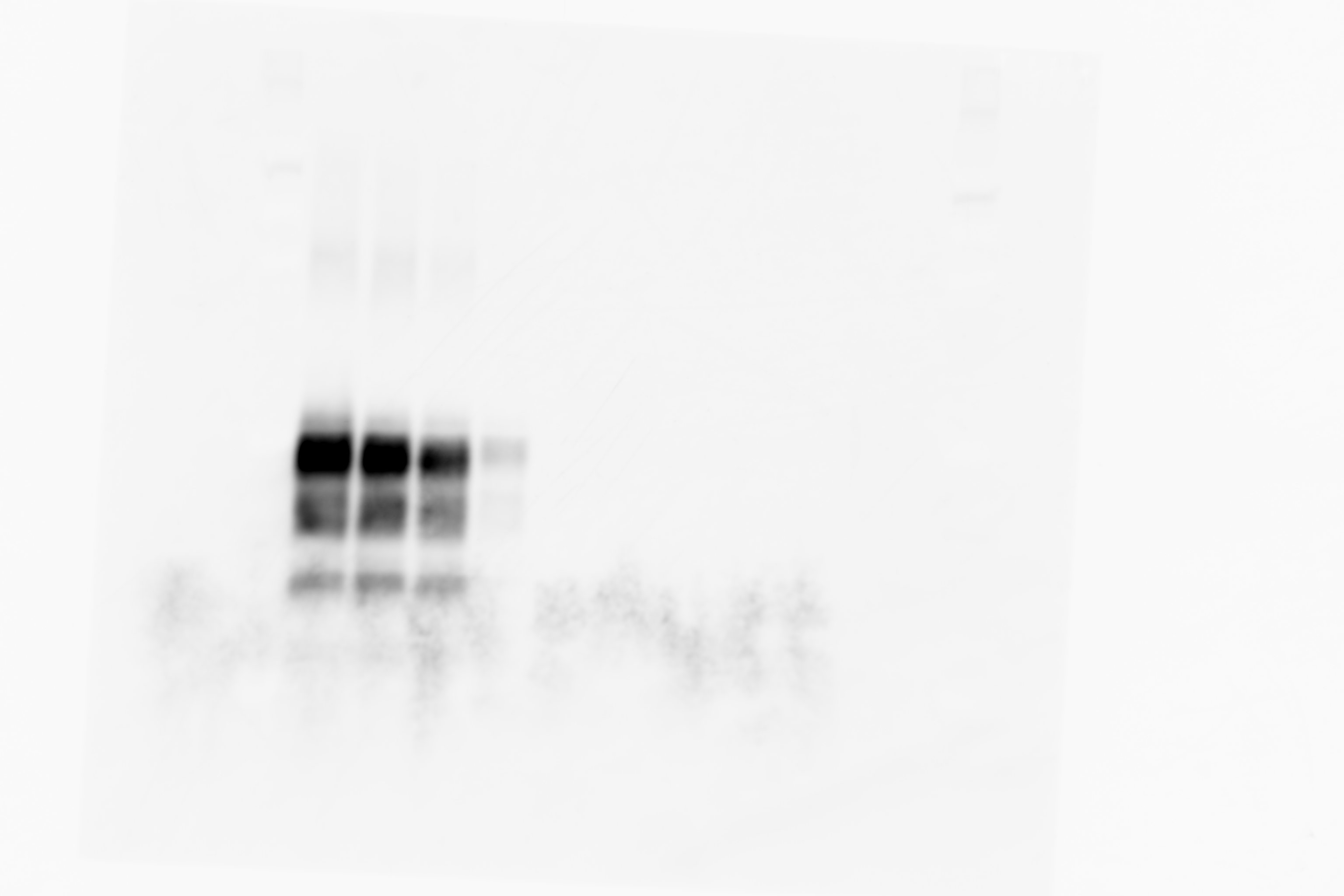

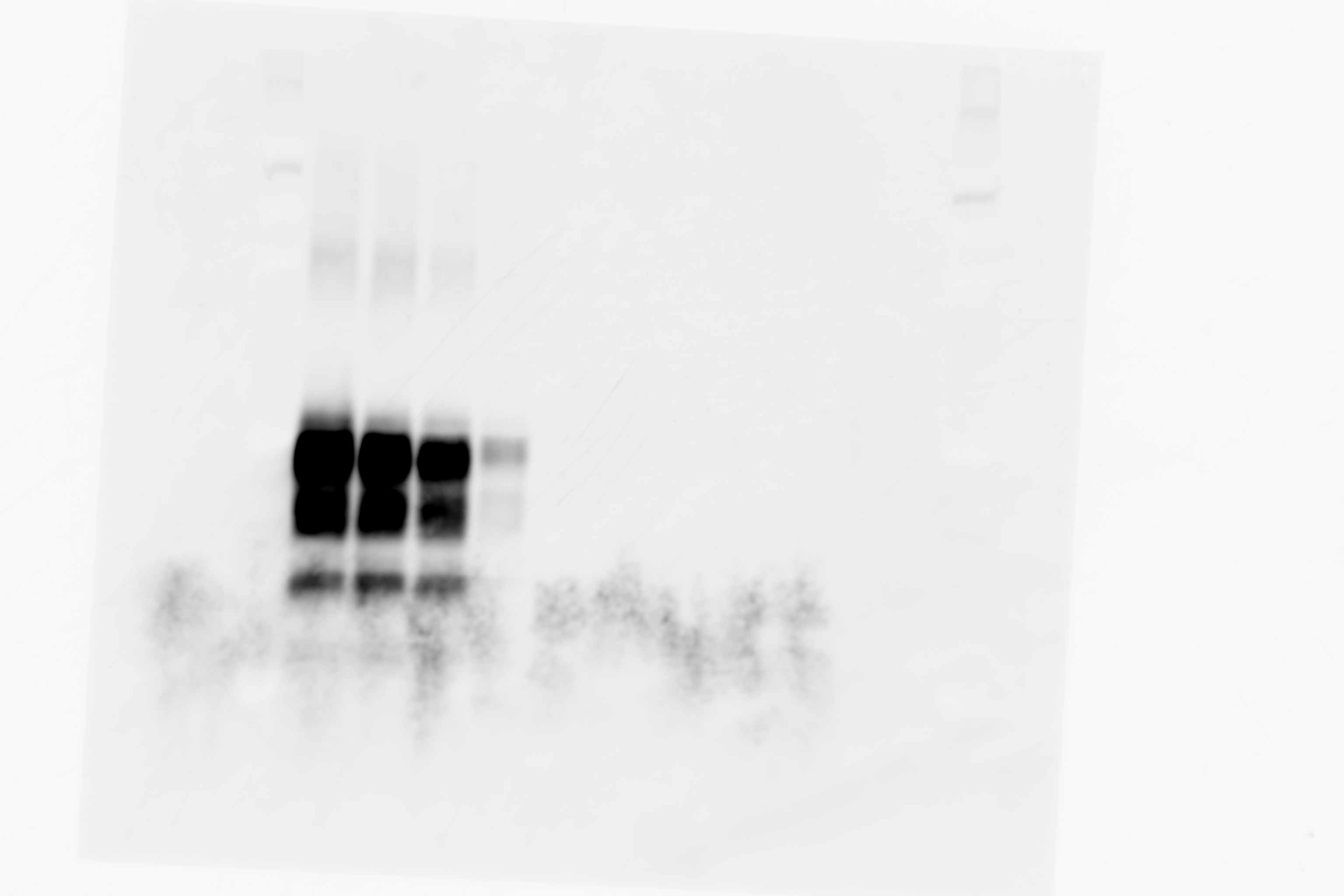

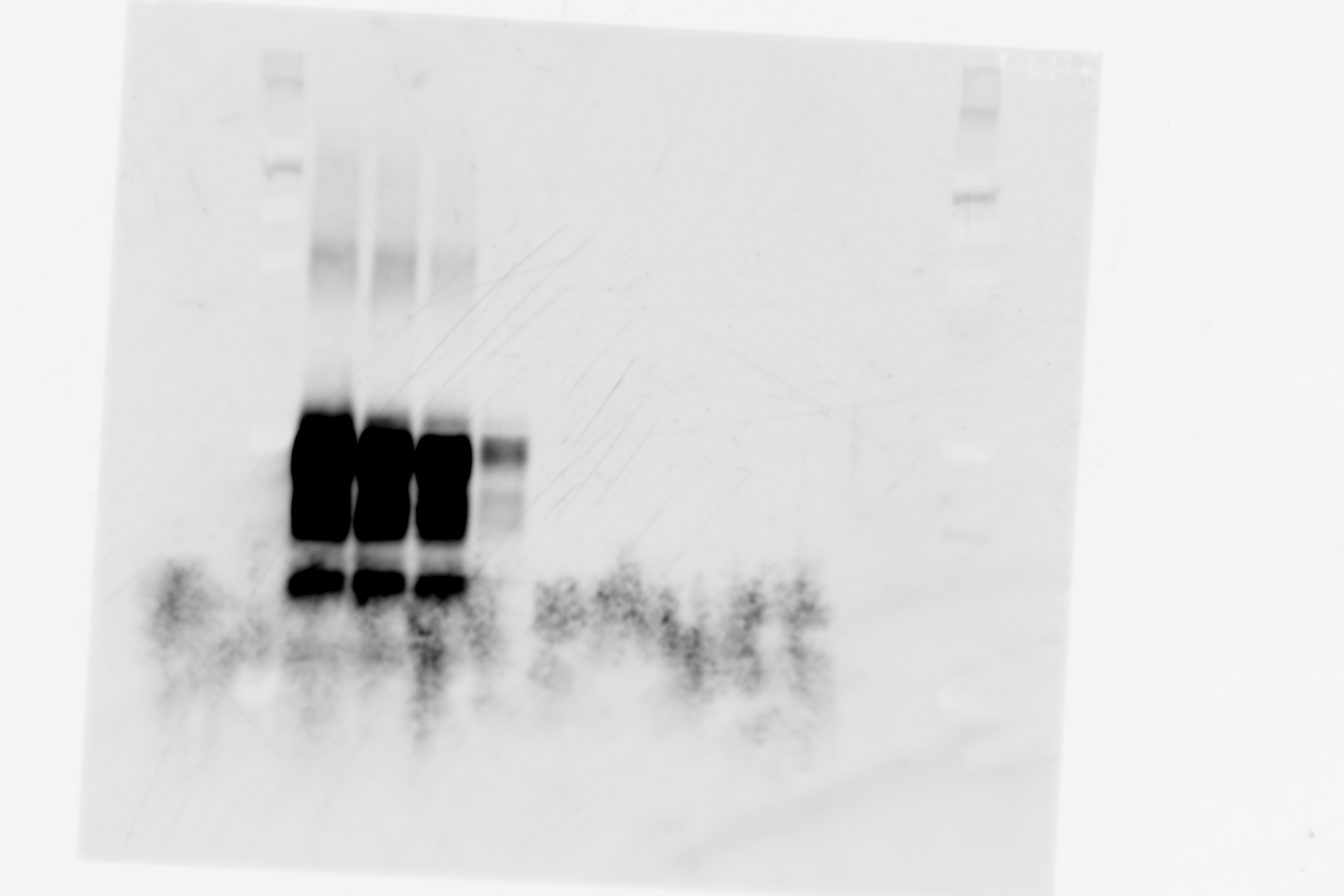

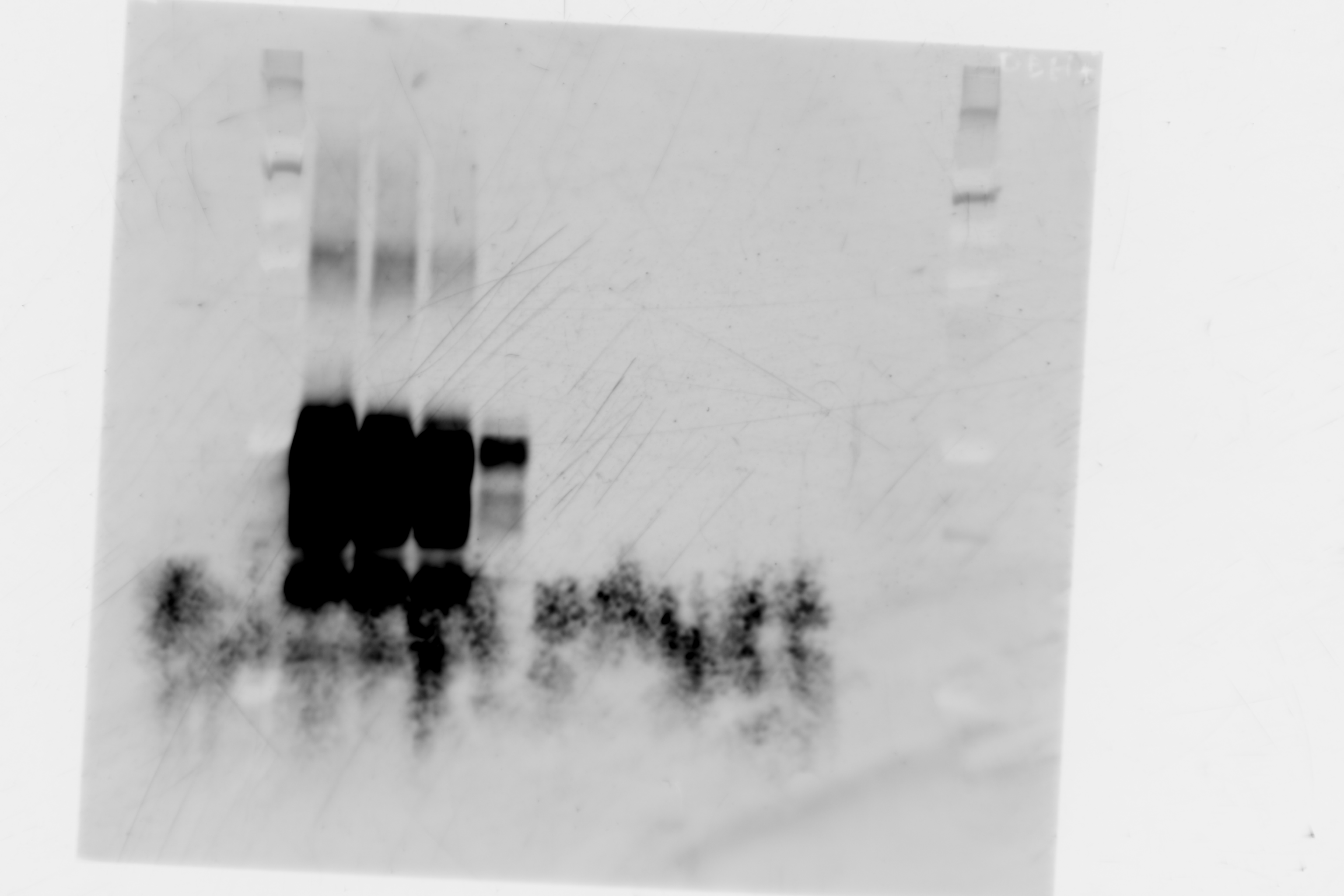

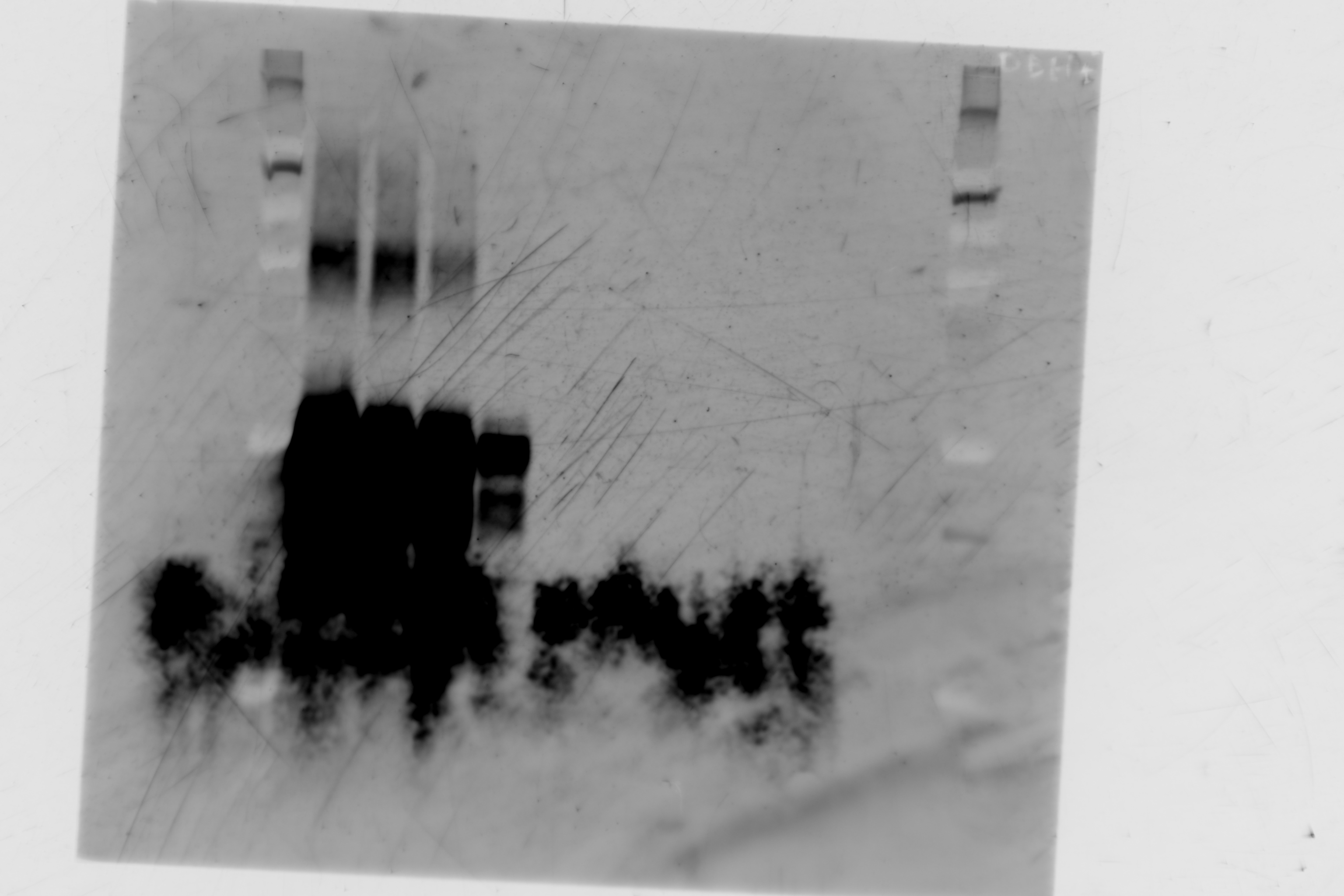

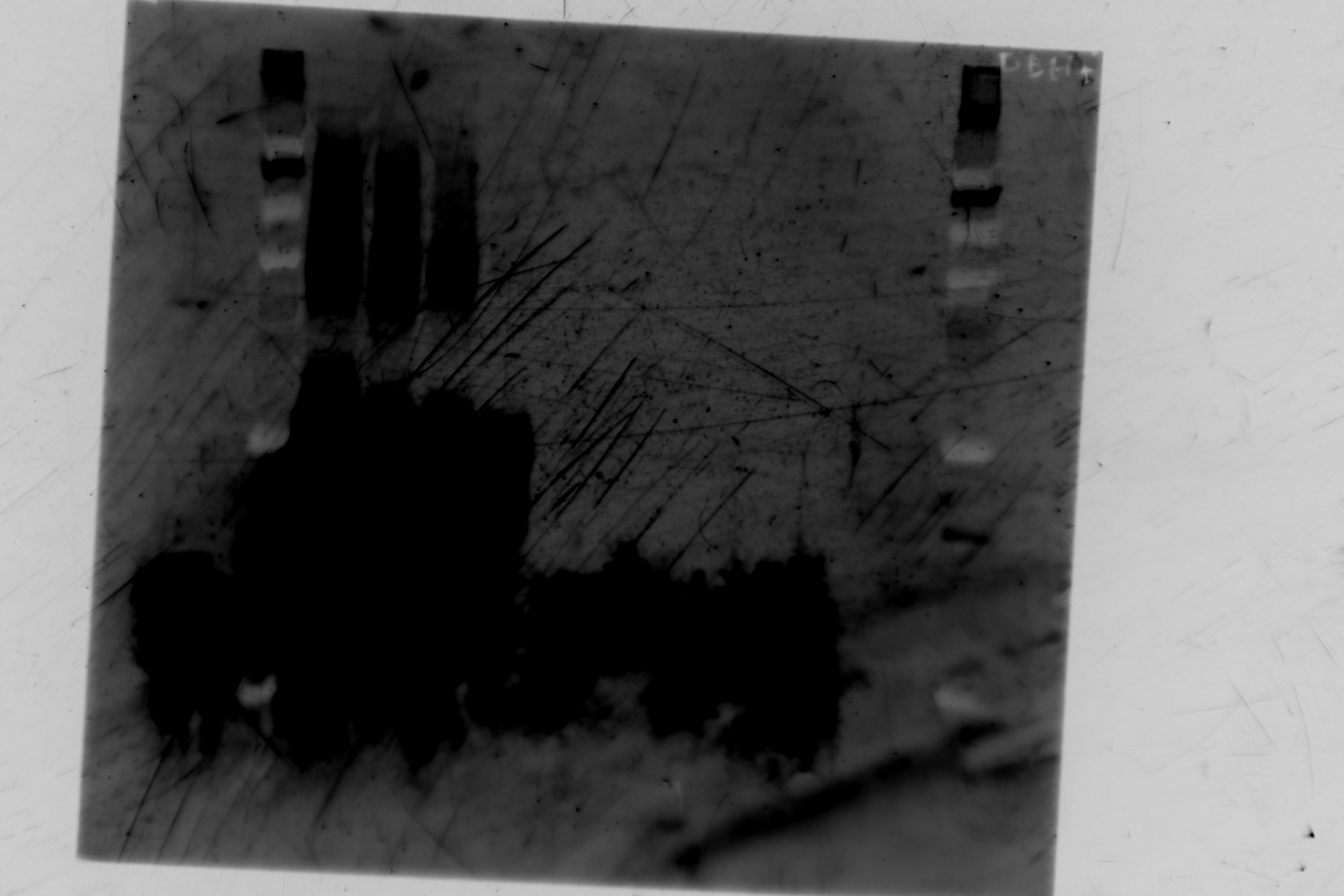

Supplement: Supplementary file 1 — Additional file 1: Supplementary Table 1. Primers and annealing temperature for qRT-PCR studies. Supplementary Figure 1. Cluster dendrograms of gene expression profiles across sequencing batch and disease phenotype. Dendrogram prepared using log-transformed counts per-million. The clustering analysis considered all genes in the data, not just those affected by treatment. Supplementary Figure 2. Heatmap depicting the VST normalized abundances of the 40 predominant bacterial species detected in neural deer tissue. Species are ranked by abundance, while columns (samples) are clustered by similarity (Ward). Heatmap prepared in R using the pheatmap package. Supplementary Figure 3. Multiple exposures of Fig. 1. [file 12864_2022_8306_MOESM1_ESM.docx]
